# Construction of lymph nodes-targeting tumor vaccines by using the principle of DNA base complementary pairing to enhance anti-tumor cellular immune response

**DOI:** 10.1186/s12951-024-02498-1

**Published:** 2024-05-08

**Authors:** Yongchao Zha, Li Fu, Zonghua Liu, Jiansheng Lin, Linghong Huang

**Affiliations:** 1 Department of Biomedical Engineering, Jinan University, Guangzhou, 510632 China; 2 Department of Anatomy, Hunan University of Chinese Medicine, Changsha, China

**Keywords:** Tumor immunotherapy, Tumor vaccines, Lymph node targeting, Cellular immunity

## Abstract

Tumor vaccines, a crucial immunotherapy, have gained growing interest because of their unique capability to initiate precise anti-tumor immune responses and establish enduring immune memory. Injected tumor vaccines passively diffuse to the adjacent draining lymph nodes, where the residing antigen-presenting cells capture and present tumor antigens to T cells. This process represents the initial phase of the immune response to the tumor vaccines and constitutes a pivotal determinant of their effectiveness. Nevertheless, the granularity paradox, arising from the different requirements between the passive targeting delivery of tumor vaccines to lymph nodes and the uptake by antigen-presenting cells, diminishes the efficacy of lymph node-targeting tumor vaccines. This study addressed this challenge by employing a vaccine formulation with a tunable, controlled particle size. Manganese dioxide (MnO_2_) nanoparticles were synthesized, loaded with ovalbumin (OVA), and modified with A_50_ or T_20_ DNA single strands to obtain MnO_2_/OVA/A_50_ and MnO_2_/OVA/T_20_, respectively. Administering the vaccines sequentially, upon reaching the lymph nodes, the two vaccines converge and simultaneously aggregate into MnO_2_/OVA/A_50_-T_20_ particles through base pairing. This process enhances both vaccine uptake and antigen delivery. In vitro and in vivo studies demonstrated that, the combined vaccine, comprising MnO_2_/OVA/A_50_ and MnO_2_/OVA/T_20_, exhibited robust immunization effects and remarkable anti-tumor efficacy in the melanoma animal models. The strategy of controlling tumor vaccine size and consequently improving tumor antigen presentation efficiency and vaccine efficacy via the DNA base-pairing principle, provides novel concepts for the development of efficient tumor vaccines.

**Graphical Abstract:**

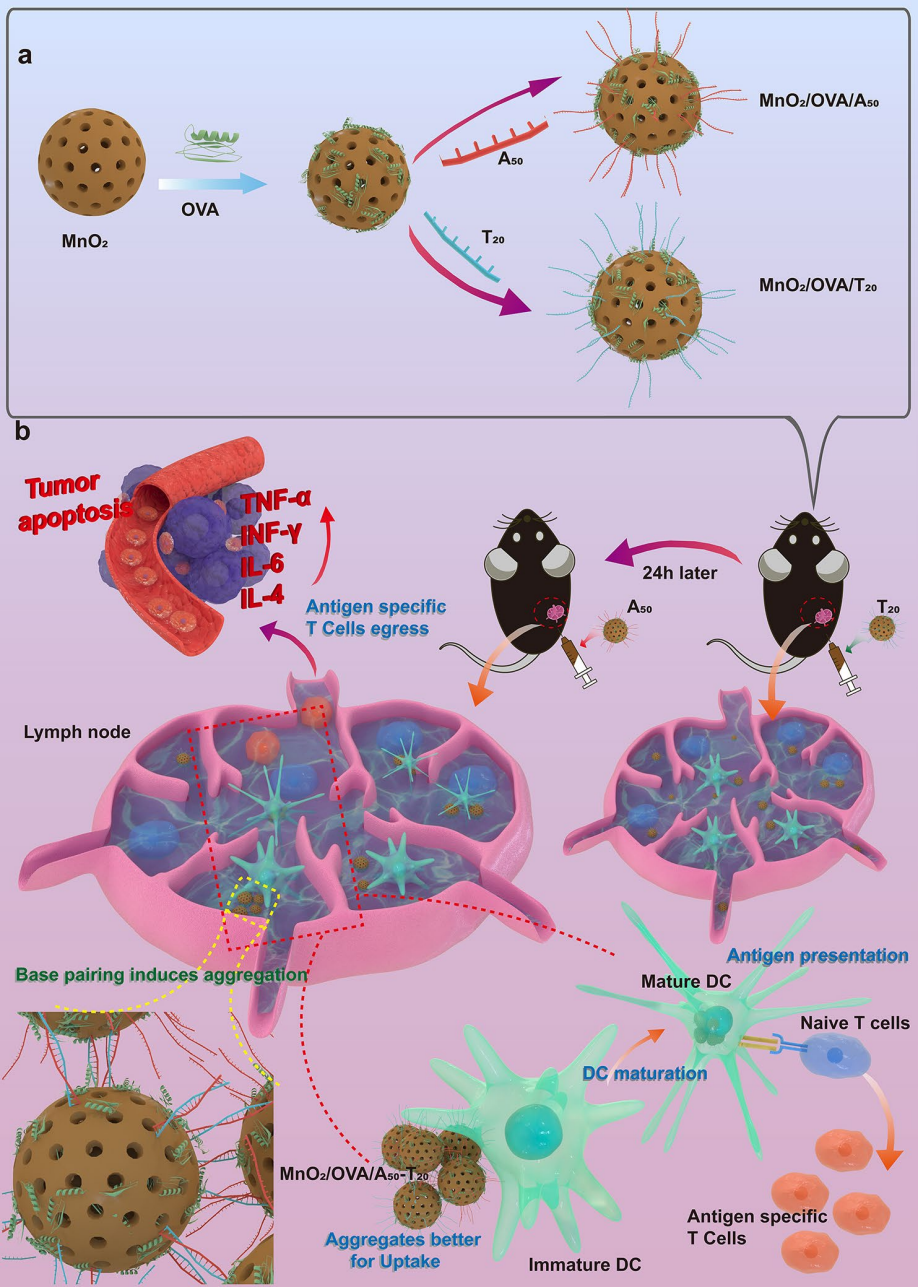

**Supplementary Information:**

The online version contains supplementary material available at 10.1186/s12951-024-02498-1.

## Introduction

In recent years, tumor immunotherapy has revolutionized the conventional paradigm of tumor treatment [1, 2]. Notably, tumor vaccines have gained growing interest owing to their outstanding ability to initiate tumor-specific immune responses and establish enduring immune memory [3, 4]. Nonetheless, current tumor vaccines usually cannot trigger efficient cellular immune responses and do not meet the demands of effective tumor therapy [5]. Low antigen presentation efficiency in the process of the intricate immune response to tumor vaccines is deemed a critical bottleneck that constrains the magnitude of the vaccine’s efficacy [6, 7]. The scarcity of antigen-presenting cells (APCs) in the peripheral tissues at conventional vaccination sites seriously hampers the capture and presentation of tumor antigens. Consequently, the administered tumor vaccines primarily passively diffuse to the adjacent draining lymph nodes, where they can be captured by the resident APCs, realizing antigen presentation [8, 9]. Lymph nodes house diverse immune cells, including APCs, B lymphocytes, T lymphocytes, and natural killer cells. They serve as vital organs for immune surveillance, regulation, and activation [10]. Notably, lymph nodes harbor a substantial population of phagocytic dendritic cells (DCs) known for their proficiency in cross-presentation [11]. This refers to presenting exogenous antigens to CD8^+^ T cells via the major histocompatibility complex I (MHC I) pathway, leading to the production of the cytotoxic T lymphocytes (CTLs) that specifically kill tumor cells [12]. More and more research substantiates that the tumor vaccines directed towards lymph nodes significantly amplify the immune response [13–16]. Therefore, the targeted delivery of tumor vaccines to lymph nodes and their uptake by APCs are pivotal factors for achieving effective antigen presentation and further triggering potent vaccine effects.

Nonetheless, the distinctive and intricate physiological structure of lymph nodes imposes specific criteria on the hydrodynamic size of vaccines. It has been found that particles exceeding 100 nm tend to become entrapped in the interstitial matrix. Conversely, particles smaller than 10 nm readily access the bloodstream via capillaries. Only those ranging from 10 to 100 nm can access the lymphatic circulation through the aqueous channels between capillaries and capillary lymphatics [17–19]. This facilitates effective vaccine drainage to the lymph nodes. Besides lymph node drainage, a crucial consideration is whether the vaccines reaching the lymph nodes can be promptly engulfed by APCs. Otherwise, the vaccines may pass through the subcapsular sinus to the outer regions of the lymph nodes and exit via efferent lymphatic vessels [20, 21]. Intriguingly, the endocytic behavior of APCs also displays a particle size preference [22]. However, different to the 10–100 nm size range necessary for lymph node drainage, APCs prefer to phagocytose particles within the 100–500 nm size range [23, 24]. Thus, the nanovaccines satisfying the criteria for both lymph node drainage and APC endocytosis hold promising potential. Recently, researchers have endeavored to address the challenges related to the particle size design of lymph node-targeted vaccines. For instance, Wang et al. employed photothermally induced phase-change materials to achieve in-situ alteration of vaccine particle size within the lymph nodes. This simultaneously improved lymph node drainage and APC uptake [25]. Qin et al. used a bioorthogonal reaction to improve vaccine targeting by first delivering a target inside the lymph node by albumin hitchhiking, and then the vaccine could be successfully targeted to the lymph node by an in vivo click reaction [26]. To date, the granularity paradox concerning targeted delivery to lymph nodes and subsequent uptake by APCs persists as a critical challenge in the development of efficient tumor vaccines.

To circumvent the need for intricate synthesis processes or additional therapeutic interventions, we advocate a streamlined approach aimed at concurrently facilitating lymph node drainage and bolstering APCs uptake within the lymph nodes, thereby eliciting a potent tumor-specific cellular immune response. Firstly, we synthesized hollow mesoporous manganese dioxide (MnO_2_) nanoparticles with an approximate particle size of about 90 nm through a hard template approach. The substantial specific surface area of these nanoparticles allowed loading of the model tumor antigen ovalbumin (OVA), resulting in the formation of MnO_2_/OVA nanoparticles. To boost the uptake by APCs within the lymph nodes, DNA single strands A_50_ and T_20_ adsorbed onto the MnO_2_/OVA nanoparticles, yielding MnO_2_/OVA/A_50_ and MnO_2_/OVA/T_20_, respectively. Subsequently, both of the nanoparticles were administered sequentially via subcutaneous inoculation by simple time-interval injections. This facilitated the entry of the vaccines into the lymphatic circulation in a state of small particle size. Upon reaching the lymph nodes, MnO_2_/OVA/A_50_ and MnO_2_/OVA/T_20_ nanoparticles converged and aggregated via the complementary binding between A_50_ and T_20_. This aggregation augments the vaccines’ particle size and facilitates their uptake by APCs within the lymph nodes. Furthermore, following internalization by APCs, the MnO_2_ within the vaccines underwent gradual degradation to Mn^2+^ within the mildly acidic environment of lysosomes, thus serving as an immune adjuvant. The immune response and anti-tumor efficacy of the MnO_2_/OVA/A_50_ -T_20_ combination vaccine were assessed through in vitro cellular assays, immunization assays, and a melanoma-bearing animal test, respectively. This innovative design strategy, capable of concurrently facilitating lymph node drainage and augmenting APC antigen uptake, can guide the development of efficient lymph node-targeting tumor vaccines.

## Materials and methods

### Materials

Tetraethyl orthosilicate (TEOS), ethanol, ammonia, potassium permanganate (KMnO_4_), and sodium carbonate (Na_2_CO_3_) were purchased from Macklin (China). Ovalbumin (OVA) was obtained from Sigma (USA). OVA-Cy5.5 was obtained from QIYUE BIOLOGY(China). DNA single strands A_50_ and T_20_ were brought from Tsingke Biotech (China). Enhanced CCK-8 kit, Lyso-Tracker green fluorescent dye, 4’,6-diamidino-2-phenylindole (DAPI) and BCA protein assay kit were purchased from Beyotime (China). Roswell Park memorial institute (RPMI) 1640 culture medium, dulbecco’s modified eagle medium (DMEM), and fetal bovine serum (FBS) brought from Procell (China). Anti-CD11c-APC, anti-MHC II-PE, anti-MHC I-PE, anti-CD80-Cy5.5, anti-CD86-FITC, FITC-anti-CD4, Cy5.5-anti-CD8a, PE-anti-CD44, APC-anti-CD62L and APC-anti-CD3 antibodies were supplied by BioLegend (USA). Enzyme-linked immunosorbent assay (ELISA) Kits for INF-γ, TNF-α, IL-4, and IL-6 were also purchased from BioLegend (USA). In addition, all animal experiments were determined eligible for the study and were approved by the Ethics Committee of Jinan University.

### Synthesis and characterization of MnO_2_

The MnO_2_ nanoparticles were synthesized by modifying previously published methods [27]. To begin, silica nanoparticles (SiO_2_) were synthesized as a hard template using the widely adopted Stöber method. Ethanol (25 mL), deionized water (0.5 mL), and ammonia (1.8 mL) were mixed in a 50 mL flask under uniform stirring. After that, TEOS (0.75 mL) was added into the flask, and the reaction was continued for 24 h at 40 ℃. The resulting SiO_2_ nanoparticles were collected by centrifugation (10,000 rpm, 20 min) and washed repeatedly. The obtained SiO_2_ nanoparticles were dispersed in deionized water (100 mg, 1 mg/ mL). KMnO_4_ solution (10 mg/mL, 75 mL) was then added dropwise to the above suspension and stirred for 6 h. The resulting nanoparticles were collected by centrifugation (10,000 rpm, 20 min) and etched with Na_2_CO_3_ solution (2 M) at 60 ℃ for 16 h to obtain the MnO_2_ nanoparticles. The morphology structures of MnO_2_ nanoparticles were observed by the TEM (JEOL TEM-1210, Japan). UV-Vis absorption spectra were measured by a UV-2550 spectrophotometer (SHIMADZU, Japan). FT-IR spectra were recorded by a FT-IR spectrophotometer (VERTEX 70). Size and Zeta potential were detected by the dynamic light scattering (DLS) (Zetasizer Nano ZS, UK).

### Preparation of Alum/OVA, MnO_2_/OVA, MnO_2_/OVA/A_50_ and MnO_2_/OVA/T_20_

The Alum/OVA vaccine was prepared by mixing 0.25 mL aluminum adjuvant with 0.1 mL OVA (3 mg/mL) and diluted with saline into a total volume of 1 mL. The MnO_2_/OVA vaccine formulation was prepared by dispersing 1 mg MnO_2_ nanoparticles in 0.9 mL saline, adding 0.1 mL OVA (3 mg/mL) solution, and mixing for 4 h. After centrifugation, the free OVA in supernatant was detected with a microplate reader (BIOTEK, USA). Subsequently, 0.5 mL MnO_2_/OVA suspension was added to 10 µL A_50_ or T_20_ (100 µM) and continued stirring for 2 h to obtain MnO_2_/OVA/A_50_ or MnO_2_/OVA/T_20_ vaccine formulation. The morphology structures of the MnO_2_/OVA/DNA nanoparticles were observed by the TEM. The UV-Vis absorption spectra of MnO_2_, MnO_2_/OVA/A_50,_ and MnO_2_/OVA/T_20_ were measured by a UV-2550 spectrophotometer (SHIMADZU, Japan). The elemental analysis of nanoparticles was detected using an energy dispersive X-ray spectrometry (EDS) in combination with a high angle annular darkfield scanning transmission electron microscopy (HAADF-STEM, JEM 2100 F, Japan).

### In vitro cytotoxicity and degradation evaluation

Firstly, DC 2.4 cells were seeded in a 96-well plate at a density of 10^4^ cells/well and cultured in an incubator for 24 h. After washing with PBS, the cells were exposed to different concentrations of MnO_2_, MnO_2_/OVA, MnO_2_/OVA/A_50_, or MnO_2_/OVA/T_20_ (20, 40, 60, 80, 100 µg/mL) for 24 h. Finally, after washing with PBS, 100 µL of 10% CCK-8 reagent was added to each well to detect cell viability.

To evaluate the degradation performance of MnO_2_, DC2.4 cells were seeded in 12-well plates, and then the nano-vaccine (concentration of MnO_2_: 20 µg/mL) was added and co-cultured with the cells for 48 h. Finally, the cells were lysed and centrifuged, and the supernatant was taken to determine the intracellular Mn^2+^concentration by the ICP.

### In vitro antigen uptake and subcellular co-localization experiment

The DC2.4 cells were seeded into a 24-well plate (1 × 10^5^ cells/well) and incubated for 24 h. After washing with PBS, the cells were incubated with free OVA-cy5.5, MnO_2_/OVA-cy5.5, MnO_2_/OVA-cy5.5/A_50_, MnO_2_/OVA-cy5.5/T_20_, or MnO_2_/OVA-cy5.5/A_50_-T_20_ (concentration of MnO_2_: 20 µg/mL, OVA: 6 ug/mL, A_50_/T_20_: 0.04 μM) for 6 h. Finally, the cells were digested with trypsin, washed with PBS, and analyzed by flow cytometry. In addition, DC2.4 cells were seeded in cell culture dishes (5 × 10^4^ cells/dish), and incubated for 24 h. Then, the cells were incubated for 6 h with free OVA-cy5.5, MnO_2_/OVA-cy5.5, MnO_2_/OVA-cy5.5/A_50_, MnO_2_/OVA-cy5.5/T_20_, or MnO_2_/OVA-cy5.5/A_50_-T_20_ formulations. Subsequently, the cells were incubated for 2 h with the Lyso-Tracker green fluorescent dye and then fixed for 20 min. Finally, the cells were stained with DAPI dye for 5 min and observed with a confocal laser scanning microscope (CLSM, Zeiss, Germany).

### Detection of lysosome integrity

The DC2.4 cells were seeded and incubated in 24-well plates (5 × 10^4^ cells/well) for 24 h. Next, the cells were stained for 1 h with 5 µg/mL acridine orange dye solution. After removing the dye, the cells were cultured for 24 h with OVA, MnO_2_/OVA, MnO_2_/OVA/A_50_, MnO_2_/OVA/T_20_, or MnO_2_/OVA/A_50_-T_20_ formulations. The distribution of acridine orange in the cells was observed with a fluorescence microscope (DMRA2, Leica, Germany).

### In vitro maturation and cross-presentation tests of BMDCs

The Bone marrow-derived dendritic cells (BMDCs) were obtained using a previously reported method [28]. In brief, the bone marrow cells were obtained from the tibia and femur of C57BL/6 mice (4–6 weeks) under aseptic conditions. The remaining cells were collected after lysis of red blood cells with red blood cell lysis buffer and cultured in RPMI 1640 complete medium containing 10% FBS, 1% penicillin/streptomycin, GM-CSF (20 ng/mL), and IL-4 (10 ng/mL). The medium was replaced with half fresh medium every two days, and the cells were harvested on the sixth day for subsequent experiments.

To assess the effect of vaccine formulations on enhancing antigen cross-presentation and DC activation, BMDCs were seeded in low-adhesion 24-well plates at a density of 5 × 10^5^ cells/well. Then, the cells were co-cultured with free OVA, MnO_2_/OVA, MnO_2_/OVA/A_50_, and MnO_2_/OVA/T_20_ or MnO_2_/OVA/A_50_-T_20_ (OVA: 5 µg/well) for 24 h. Finally, BMDCs were collected and stained with anti-CD11c-APC, anti-MHC II-PE, anti-MHC I-PE, anti-CD80-Cy5.5, and anti-CD86-FITC, and then quantitatively analyzed using a flow cytometer. Further, in order to verify the mechanism by which MnO_2_ promotes DC maturation, we measured the level of INF-β secreted by BMDC in the presence of manganese dioxide by using an mINF-β 2.0 assay kit (InvivoGen, USA).

### In vivo evaluations of LNs targeting

To determine the optimal time for materials to reach LNs, C57BL/6 mice (6–8 weeks) were selected for subcutaneous injection of MnO_2_/OVA-Cy5.5 (*n* = 3). At 2, 6, 12, 24, and 48 h after injection, inguinal LNs near the injection site were subsequently collected and imaged ex vivo using a small animal bioluminescence imaging system (IVIS Lumina III, PerkinElmer, USA). To verify the better LNs retention ability of the MnO_2_/OVA-cy5.5/A_50_-T_20_, C57BL/6 mice (6–8 weeks) were subcutaneously injected with 50 µL Saline, OVA-Cy5.5, MnO_2_/OVA-Cy5.5, MnO_2_/OVA-Cy5.5/A_50_, MnO_2_/OVA-Cy5.5/T_20_, and MnO_2_/OVA-Cy5.5/A_50_, respectively. After 24 h, 50 µL Saline, OVA-Cy5.5, MnO_2_/OVA-Cy5.5, MnO_2_/OVA-Cy5.5/A_50_, MnO_2_/OVA-Cy5.5/T_20_, and MnO_2_/OVA-Cy5.5/T_20_ were injected again in same site. Inguinal LNs near the injection site were collected 24 h later for ex vivo IVIS imaging which was performed by an IVIS Lumina XR equipment (IVIS Lumina III, USA).

### Immunization and immune responses

The female 6–8 weeks C57BL/6 mice were randomly divided into seven groups (*n* = 6) and subcutaneously injected with OVA, Alum/OVA, MnO_2_/OVA, MnO_2_/OVA/A_50_, MnO_2_/OVA/T_20,_ and MnO_2_/OVA/A_50_-T_20_ on days 0, 1, 7, 8, 14, and 15, respectively. On day 21, the spleens were collected to prepare splenocyte suspension for subsequent detection.

Flow cytometry was used to quantitatively analyze DC maturation and T cell differentiation in the spleen of different groups of mice. Direct staining of splenocytes with anti-CD11c-FITC, anti-MHC I-PE, anti-CD86-Cy5.5, and anti-CD80-APC was used to observe the maturation of DCs in splenocytes, and the efficiency of cross-presentation of antigen. Splenocytes were stained with anti-CD3-APC, anti-CD8a-Cy5.5, and anti-CD4-FITC to determine the proportion of CD4^+^ and CD8^+^ T cells in splenocytes. To visualize the proportion of memory T cells in splenocytes, splenocytes were directly stained with FITC-anti-CD4, PerCP-Cy5.5-anti-CD8a, PE-anti-CD44, and APC-anti-CD62L, and then detected by the flow cytometer which carried out on a Beckman CytoFLEX flow cytometry (USA).

### In vivo antitumor efficacy

C57BL/6 mice (6-8weeks) were randomly divided into six groups (*n* = 6) and inoculated with B16-OVA cells (6 × 10^6^ cells/mouse) on the back of the mice, and subsequently injected with each vaccine when the tumor size was approximately 50 mm^3^, which was marked as day 0. The mice were injected with a vaccine formulation (OVA: 15 µg) on days 0, 1, 5, 6, 10, and 11. The body weight and tumor volume were monitored at one-day intervals. The tumor volume was calculated using the formula: V= (L×W^2^)/2 (where V is the tumor volume, L is the length of the tumor, and W is the width of the tumor). On day 15, the tumors, serum, LNs, spleen, and other major organs (heart, liver, lung, and kidney) were collected for further analysis. The tumor slices were stained with hematoxylin-eosin (H&E), TUNEL, Ki67, and CD8^+^ dye solutions, and observed with the fluorescence microscope. In addition, the spleens and lymph nodes were collected to prepare splenocyte and lymphocyte suspensions, respectively. The splenocytes and lymphocytes were then stained with anti-CD3-APC, anti-CD8a-Cy5.5, and anti-CD4-FITC to determine the proportion of CD4^+^ and CD8^+^ T cells. The flow cytometry was carried out on a Beckman CytoFLEX flow cytometry (USA).

### Statistical analysis

All results are presented as the mean ± S.D. from at least three independent experiments. Statistical differences between two groups were determined by Student’s t-test, and the differences among three or more groups were determined by one-way or two-way NOVA with the Bonferroni multiple comparison post-test. P values of *p < 0.05, **p < 0.01, ***p < 0.001, and ****p < 0.0001 were regarded as significant differences. Animal survival rates were compared with the log-rank test using GraphPad Prism 9.5.

## Results and discussion

### Preparation and characterization of MnO_2_/OVA/DNA

**Fig. 1:**
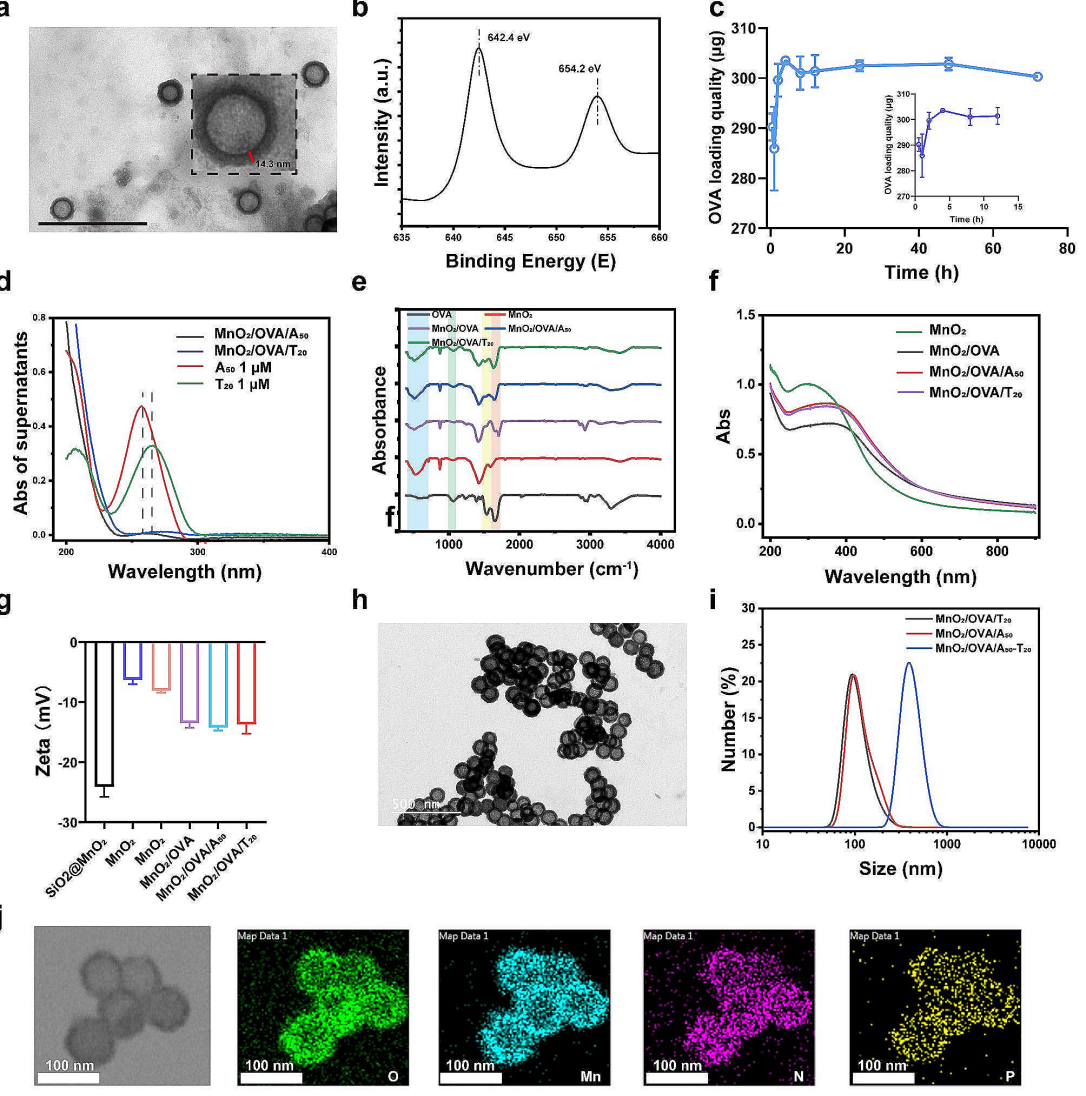
Characterizations of synthesized vaccine. **a**) TEM image of MnO_2_ nanoparticles. Scar bar: 500 nm. **b**) XPS analysis of MnO_2_ nanoparticles. **c**) The capacity of OVA by MnO_2_ nanoparticles. **d**) Changes in DNA absorption peaks in the supernatant before and after loading the MnO_2_/OVA with DNA. **e**) FT-IR spectra of different samples. **f**) UV–vis absorption spectra of different samples. **g**) Zeta potentials of the intermediate products during the preparation process of nanovaccines. **h**) TEM images of MnO_2_/OVA/A_50_ and MnO_2_/OVA/T_20_ after mixing. **i**) The size curve of MnO_2_/OVA /A_50_ and MnO_2_/OVA/T_20_ before and after mixing. **j**) HAADF-STEM images of MnO_2_/OVA /A_50_ -T_20_

Hollow manganese dioxide was synthesized by the method previously reported in the literature with a mirror change [27]. As shown in the TEM image (Fig. 1a), MnO_2_ showed a uniform spherical morphology with an average particle size of around 90 nm, and the shell thickness of MnO_2_ was uniform at about 14.3 nm. As anticipated, manganese elements were shown in the X­ray photoelectron spectroscopy (XPS) spectrum (Fig. 1b), the characteristic peaks at 654.2 and 642.4 eV corresponded to the Mn (IV) 2p_2/3_ and Mn (IV) 2p_1/2_ spin–orbit peaks of MnO_2_, indicating that MnO_2_ nanoparticles have been successfully synthesized [29, 30].

The results of the BCA kit assay showed that the OVA loading of MnO_2_ could reach 300 µg/mg within 4 h (Fig. 1c). For the loading of two different DNA single strands, the supernatants of DNA single strands (A_50_ and T_20_) before and after loading by MnO_2_/OVA nanoparticles were examined by UV-Vis spectroscopy, as shown in Fig. 1d. The characteristic absorption peaks of A_50_ and T_20_ in the supernatants disappeared after loading by MnO_2_/OVA nanoparticles, which indicated that the MnO_2_/OVA nanoparticles successfully loaded with DNA single strands. The morphologies of MnO_2_/OVA/DNA was shown in Supporting Information Figure S1a, and it can be seen that they are not significantly different from MnO_2_. In addition, FT-IR analysis showed (Fig. 1e) that MnO_2_/OVA, MnO_2_/OVA/A_50_, and MnO_2_/OVA/T_20_ showed characteristic peaks at 1645 cm^-1^ and 1524 cm^-1^ attributed to amide bonds, indicating the successful loading of OVA [31]. The characteristic peaks of MnO_2_/OVA/A_50_ and MnO_2_/OVA/T_20_ at 1067 cm^-1^ were attributed to phosphate groups, indicating the successful loading of DNA single chains [32]. Subsequently, UV-Vis spectra were recorded to distinguish the different products from material synthesis to vaccine preparation (Fig. 1f), MnO_2_ showed broad spectrum absorption at 200–600 nm. In contrast, vaccine formulation loaded with OVA and DNA single strands caused a different degree of red shift in the position of the maximum absorption peak of MnO_2_. The zeta potentials of the intermediates (Fig. 1g) of the vaccine formulations all changed significantly, indicating that the modification and loading at each step of the vaccine formulations process were successful.

To test if MnO_2_/OVA/A_50_ and MnO_2_/OVA/T_20_ could achieve base-pairing-triggered aggregation, we demonstrate the occurrence of nanoparticle aggregation phenomena after a simple mixing of the two groups of materials through TEM images (Fig. 1h), which shows that the vaccine aggregates formation. The DLS further demonstrated the change in particle size which the size increased from 91.0 to 91.3 nm to 396 nm after simple mixing (Fig. 1i). Notably, the size measured by DLS is much larger than that measured by TEM, which may be attributed to the different imaging conditions as the measurement of TEM was conducted under a dry state, while DLS measurements were in fully hydrated states [33]. The results together demonstrated that MnO_2_/OVA/A_50_ and MnO_2_/OVA/T_20_ could rapidly form aggregation with simple mixing. In addition, the results of the elemental analyses showed that MnO_2_/OVA/A_50_ and MnO_2_/OVA/T_20_ aggregated into large size particles containing the Mn, N, O, and P elements (Fig. 1j). Together, these results confirm that MnO_2_/OVA/A_50_ and MnO_2_/OVA/T_20_ could aggregate in response to A_50_ and T_20_ base pair pairing.

### In vitro assessment of cytotoxicity, cellular uptake, and antigen lysosome escape

**Fig. 2:**
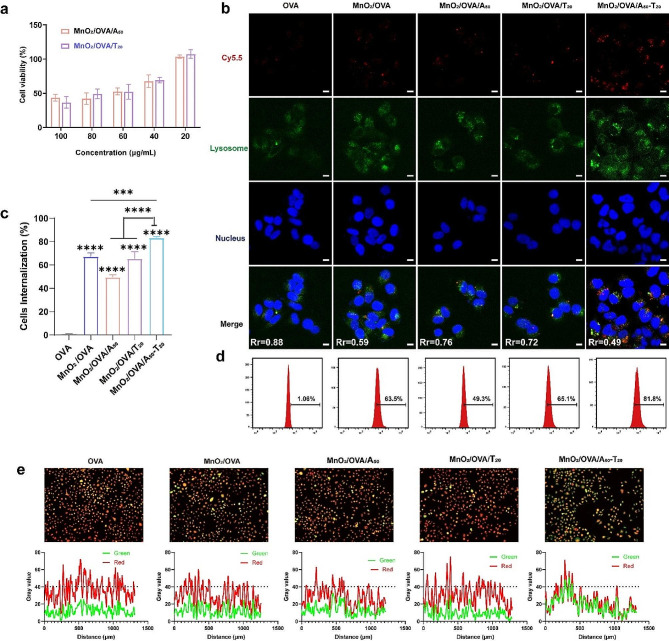
In vitro cellular assays of various vaccine formulations. **a**) Cytotoxicity assessment of MnO_2_/OVA/A_50_ and MnO_2_/OVA/T_20_ incubated with DC2.4 cells for 24 h and measured by the CCK-8 assay kit. **b**) CLSM images of DC2.4 cells after incubation with different vaccine formulations for 6 h. OVA, lysosome, and cell nucleus were respectively labeled with Cy5.5 (red), LysoTracker FITC (green), and DAPI (blue). Rr is the Pearson correlation coefficient for fluorescence co-localization of lysosomes with OVA (analyzed by ImageJ). The scale bar represents 10 μm. **c**) Corresponding mean fluorescence intensity of every group in the cellular uptake assay. **d**) Representative flow cytometry histograms for OVA uptake efficiencies of DC2.4 cells in various groups. **e**) Lysosomal integrity observation of DC 2.4 cells exposed to vaccine preparations for 24 h, and semi-quantitative analysis of fluorescence (analyzed by Image J). The significance was analyzed using a one-way analysis of variance (ANOVA). *p < 0.05, **p < 0.01, ***p < 0.001, ****p < 0.0001

An initial assessment of the biocompatibility of the vaccine is required before a comprehensive evaluation of the vaccine through various cellular and animal assays. After treating the cells with different concentrations of MnO_2_, MnO_2_/OVA, MnO_2_/OVA/A_50_, and MnO_2_/OVA/T_20_ for 24 h, the cell viability was detected by using the CCK-8 kit. As shown in Supporting Information Figure S1b and Fig. 2a, MnO_2_ exhibited slight cytotoxicity within a certain range, but the loading of OVA and DNA strands could effectively enhance the biosafety of the vaccine. Finally, 20 µg/mL was chosen as the optimal dose of MnO_2_/OVA/DNA for subsequent in vitro experiments. These results of the in vitro cytotoxicity experiments showed that the nanovaccines prepared in this study have good biosafety and can be used for further bioassays.

The effective endocytosis of nanovaccines by antigen-presenting cells plays a decisive role in activating the adaptive immune response. Therefore, Cy5.5-labeled OVA antigen was used to explore the endocytosis efficiency of different nanovaccines. The DCs were subsequently co-incubated with different vaccine groups for 6 h and the distribution of antigen in the cells was observed by CLSM. As shown in Fig. 2b, the green and red signals represented lysosomes and OVA, respectively. Due to the low uptake of free OVA by the cells, the red fluorescent signal was weaker in the OVA group and the green signal almost overlapped with the red signal, indicating that OVA failed to be released from the lysosomes into the cytoplasm. In contrast, the red fluorescence signal was significantly enhanced in the MnO_2_-containing groups, and antigen lysosome escape was also observed. This indicated that biodegradable MnO_2_ can successfully deliver antigens to the cytoplasm. Notably, the red fluorescence signal in the A-T base pair pairing group (MnO_2_/OVA-cy5.5/A_50_-T_20_) was the strongest and was found to be significantly less co-localized with the lysosomes by fluorescence intensity paving. This corroborates that the A-T base pair pairing group has higher antigen endocytosis efficiency and stronger lysosomal escape ability. In addition, as shown in Fig. 2c-d, the endocytosis efficiency of all vaccine formulations with MnO_2_ as the carrier was significantly higher than that of the free OVA group, while the endocytosis efficiency of the A-T base pair pairing group also separated significantly from the other MnO_2_ carrier groups, indicating that due to the increased particle size in the A-T base pair pairing group, it was able to be more effectively taken up by DC cells, thus may promote the adaptive immune response [25].

By evaluating the degradation behavior of the vaccine in the cell, it was found that the vaccine could reach 10% degradation rate at 48 h after cellular uptake, which provides the possibility for the vaccine to achieve lysosomal escape (Supporting Information Figure S1c). We speculate that MnO_2_ decomposes to Mn^2+^ in the micro acidic environment within the lysosome, which may lead to an increase in lysosomal osmotic pressure, disrupting the lysosome and thus promoting antigen cross-presentation. Acridine orange, a hetero-staining fluorescent dye, exhibits red fluorescence in lysosomal species and green fluorescence in the cytoplasm and can be used to detect lysosomal integrity. When the lysosomal membrane is disrupted, the intensity of red fluorescence decreases [34]. As shown in Fig. 2e, free OVA had little effect on the lysosomes, which are structurally intact and show more orange fluorescence under fluorescence microscopy. In contrast, the MnO_2_-containing group showed different degrees of diminished red fluorescence, which indicated that the structural integrity of the lysosomes might be damaged to different degrees. Among them, the red fluorescence in the A-T base pair pairing group (MnO_2_/OVA/A_50_-T_20_) was most obviously diminished, and the superimposed fluorescence photos showed yellow-green fluorescence, indicating that this group had the greatest lysosomal damage. The semi-quantitative fluorescence analysis of the photographs also showed the same trend. This may be because of the greater uptake of MnO_2_ by APCs in the A-T base pair pairing group, resulting in greater release of Mn^2+^, which promotes lysosomal rupture and antigen lysosome escape.

### In vitro evaluation of activation and antigen cross-presentation of BMDCs

**Fig. 3:**
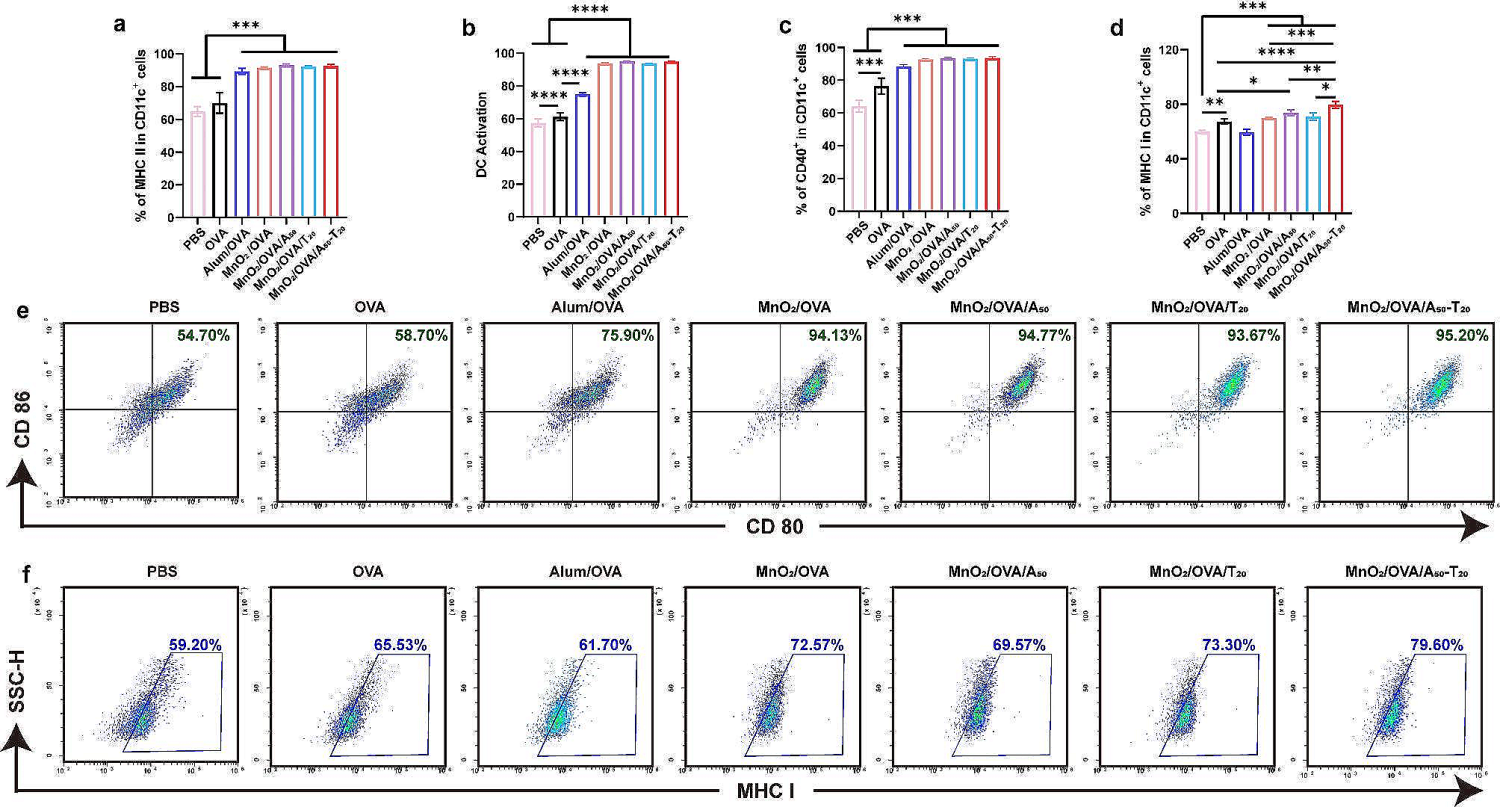
Nanovaccines efficiently activate BMDCs in vitro. The expression of **a**) MHC II, **b**) CD80 and CD86, **c**) CD40, and **d**) MHC I in CD11c^+^ BMDCs. Representative flow cytometry dot plots of **e**) DCs maturation and **f**) MHC I expressed on CD11c^+^ BMDCs in various groups (gated on CD11c^+^ cells). The representative plot dots of MHC II and CD 40 were shown in Figure S2. Error bars represent ± SD (*n* = 3). The significance was analyzed using one-way analysis of variance (ANOVA). *p < 0.05, **p < 0.01, ***p < 0.001, ****p < 0.0001

DCs are the most potent antigen-presenting cells known, capable of efficient uptake, processing, and presentation of antigens. Immature DCs become mature DCs after capturing antigens, expressing co-stimulatory molecules such as CD80, CD86, etc. When antigen escapes from the lysosome to the cytoplasm, facilitating its delivery to CD8^+^ T cells via the MHC I pathway to produce CTL, i.e., antigen cross-presentation. To demonstrate that the vaccine stimulates DC maturation and promotes antigen cross-presentation, BMDCs were extracted from mice and co-cultured with the vaccine formulations for 24 h. Then, the expression of OVA-specific MHC I, MHC II, CD80, and CD86 molecules of BMDCs were analyzed, and the results were shown in Fig. 3 and Supporting Information Figure S3 (flow cytometry gating strategy was shown in Supporting Information Figure S2). Compared with the OVA group compared with the OVA group, the MnO_2_-containing group exhibited significant upregulation of MHC II (Supporting Information Figure S3a & Fig. 3a), and compared with the Alum/OVA group, the MnO_2_-containing group exhibited significant upregulation of CD80 and CD86 molecules (Fig. 3b and e) and CD40 molecules (Supporting Information Fig. 3c & Figure S3b), which indicated that MnO_2_ effectively stimulated the maturation of BMDCs and promoted the antigen presentation. Notably, the A-T base pair pairing group showed the highest expression of MHC I molecules (Fig. 3d and f), which was consistent with the previous results of cellular endocytosis and antigen lysosome escape experiments, suggesting that A_50_ and T_20_ pairing promotes vaccine aggregation to elicit stronger antigen cross-presentation based on its higher endocytosis efficiency. Manganese is a nutritious inorganic trace element required for a variety of physiological processes. Jiang et al. [35, 36] found that Mn^2+^ in the cytoplasm can play an alarming role, activate the cGAS-STING cascade, promote the production of large amounts of interferon type I, and enhance the activation of DC and the cross-presentation reaction of antigens. Therefore, we further examined the concentration of type I interferon IFN-β in the DC supernatant after incubation with MnO_2_ nanovaccines, and the results are shown in Supporting Information Figure S4. MnO_2_ increased the Mn^2+^ content in the cytoplasm, and significantly increased the concentration of IFN-β in the DC supernatant, which is an important reason for the enhancement of DC maturation.

### In vivo LNs targeted delivery

**Fig. 4:**
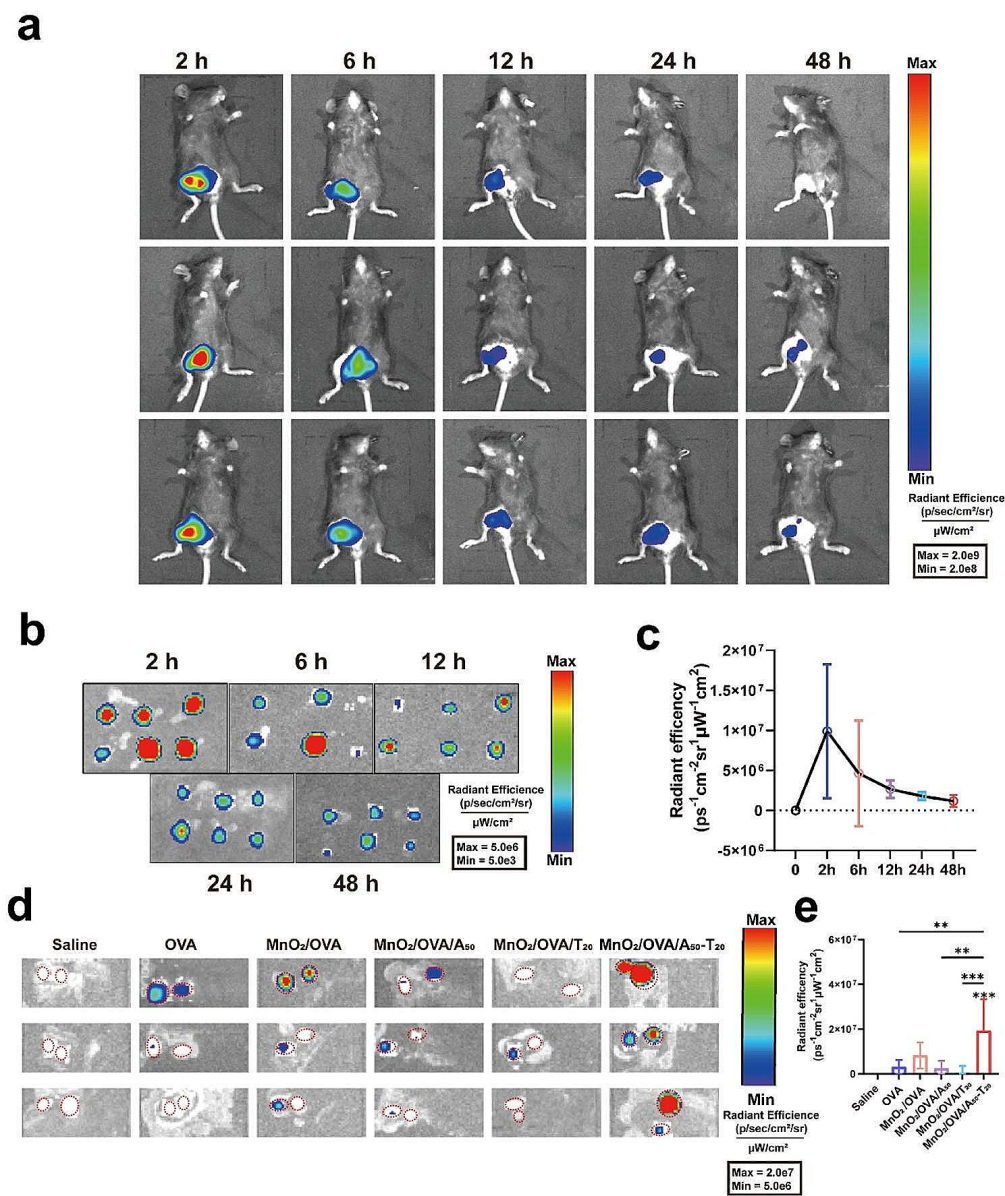
In vivo systemic distribution of nanovaccines. **A**) In vivo fluorescence imaging of mice at different times (2, 6, 12, 24, and 48 h) after subcutaneously injected MnO_2_/OVA (100 µL per mouse). OVA was labeled with Cy5.5. **b**) IVIS fluorescence imaging of the isolated inguinal LNs from C57BL/6 mice (*n* = 3) at different time points after MnO_2_/OVA (100 µL per mouse) administration. **c**) Quantification of the accumulated fluorescence in LNs over time. **d**) IVIS images and **e**) quantification of the fluorescence in LNs from the mice (*n* = 3) injected with saline, free OVA, MnO_2_/OVA, MnO_2_/OVA/A_50_, MnO_2_/OVA/T_20_ or MnO_2_/OVA /A_50_-T_20_. Error bars represent ± SD (*n* = 3). The significance was analyzed using a one-way analysis of variance (ANOVA). *p < 0.05, **p < 0.01, ***p < 0.001, ****p < 0.0001

For passive LNs targeting vaccines, the LNs drainage capacity and LNs retention capacity of the vaccine are most critical. Next, the LN’s targeting ability of the vaccine was examined using the IVIS imaging system. Considering that factors affecting lymph node drainage (size, shape and zeta potential) were not significantly different between MnO_2_/OVA and MnO_2_/OVA/DNA nanoparticles, the MnO_2_/OVA-Cy5.5 vaccines were selected as the study subject and injected subcutaneously into the right and left groin of C57BL/6 mice. The mice were then performed live imaging at 2, 6, 12, 24, and 48 h, after which the inguinal LNs were isolated for fluorescence imaging to observe the accumulation of fluorescence at the LN sites. As shown in Fig. 4a, the fluorescence at the injection site gradually decreased with time and disappeared at 24 h, indicating that the vaccine gradually migrated passively, possibly to the LNs. Therefore, the LNs were isolated from the mice and their fluorescence intensity was observed at each time point. As shown in Figure b-c, the fluorescence of the LN was strongest at 2 h after injection, indicating that the vaccine reached the highest amount of LN at this time. Subsequently, the fluorescence intensity of the LNs began to diminish with time. This corroborates the view reported in the literature that due to the weak retention capacity of LNs, the conventional materials enter the LNs merely by passing through, and cannot be effectively endocytosed by APCs.

Next, for LNs-targeted vaccines administered in two doses, the timing of the second dose is critical for vaccine efficacy. Based on the results of LNs ex vivo imaging, an interval of 2 h should have been chosen as the time of the second vaccination. However, considering that a large amount of the first dose of vaccine was still left at the injection site, the second dose of vaccine at this time might cause the vaccine to accumulate at the injection site and affect the efficiency of its LN drainage. Therefore, we chose 24 h as the time interval for the second vaccine injection and took this as the standard for the complete two vaccine doses in this study (Supporting Information Figure S5). The inguinal LNs were removed for fluorescence imaging 24 h after the second vaccine dose, as shown in Fig. 4d-e. The fluorescence signals in the LNs of the conventional vaccine group in which the nanovaccines could not accumulate in the LNs were at a similar and lower level. The A-T base pair pairing group (MnO_2_/OVA-cy5.5/A_50_-T_20_), on the other hand, showed strong fluorescence, indicating that the MnO_2_/OVA-cy5.5/A_50_-T_20_ group had a stronger capacity to detain in LNs. In addition, no significant differences were found in the accumulation of vaccines in the livers and spleens (Supporting Information Figure S6). Taken together, the experimental results showed that the 1st particle dose can still be compounded with 2ed particle dose, increasing the size of the nanovaccines in the LNs and significantly increasing the amount of vaccine accumulation within the lymph nodes which indicates the strategy of inducing vaccine aggregation in LNs through A-T base pair pairing to enhance vaccine uptake by APCs has achieved preliminary success.

### Enhanced in vivo cellular immune responses

**Fig. 5:**
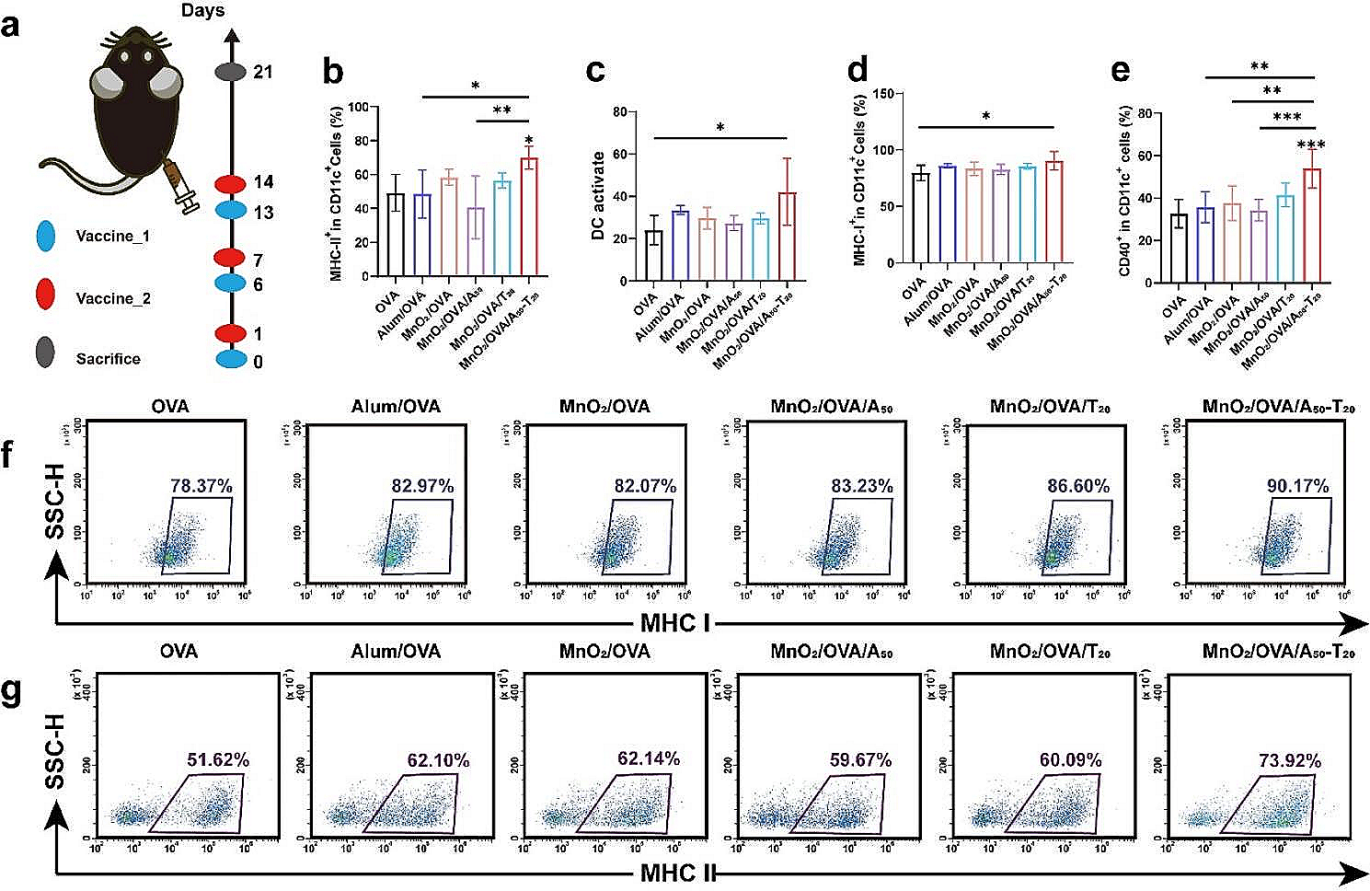
The evaluation of immune response after C57BL/6 mice were immunized with various vaccine formulations three times. Schematic illustration of the process of s. c. injection of nano-vaccines in preventive immune mode. **b**-**e**) Percentage of **b**) MHC II, **c**) DCs maturation, d) MHC I, **e**) CD40 expressed on CD11c^+^. Representative flow cytometry plots of f) MHC II and **g**) MHC I in spleen harvested from immunized mice. The representative plot dots of DC maturation and CD40 were shown in Figure S5. Error bars represent ± SD (*n* = 6). The significance was analyzed using a one-way analysis of variance (ANOVA). *p < 0.05, **p < 0.01, ***p < 0.001, ****p < 0.0001

**Fig. 6:**
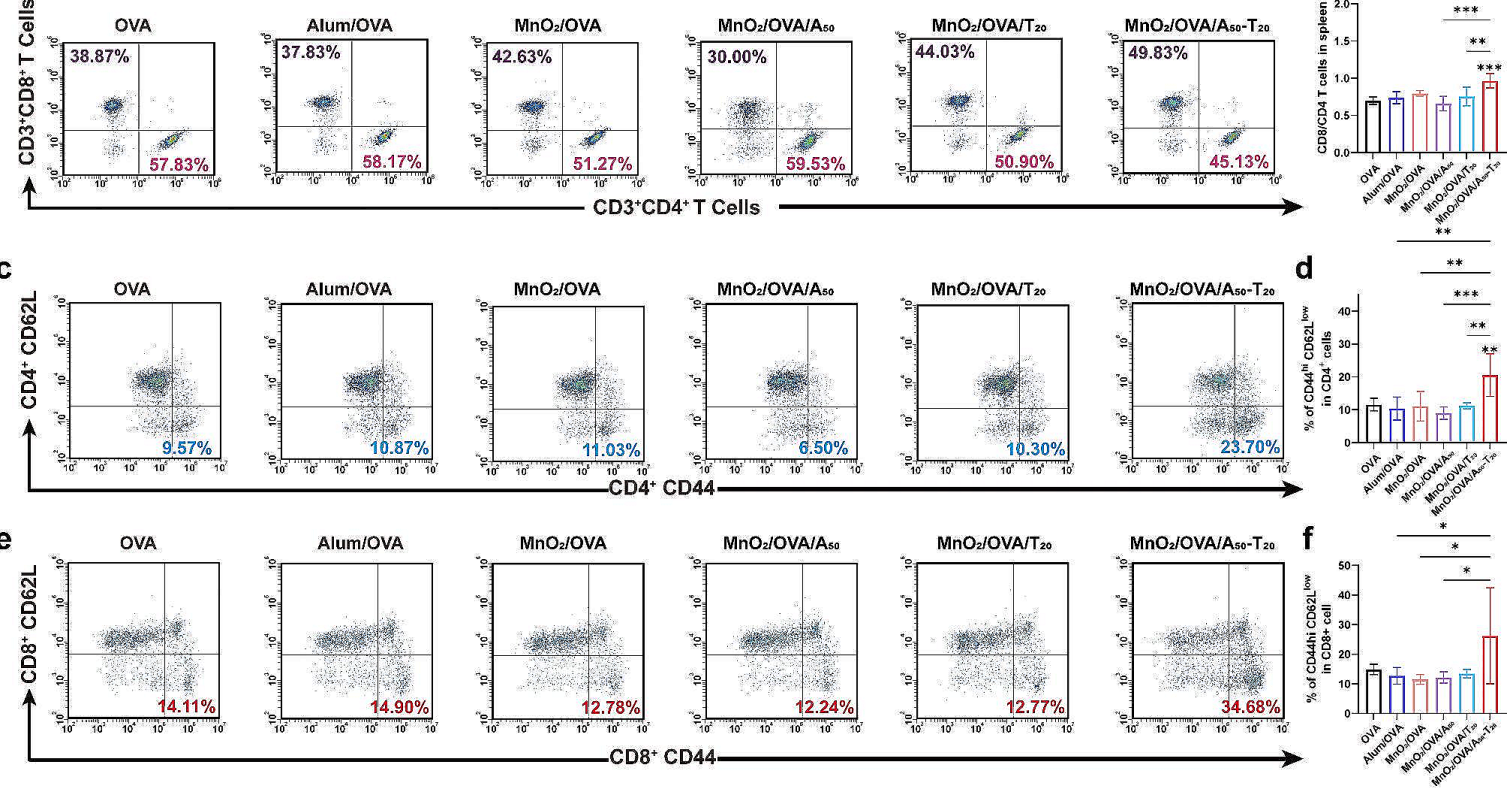
The assessment of immune memory effects after the mice were immunized with various vaccine formulations three times. **a**) Representative flow cytometry dot plots of CTLs (gated on CD3^+^ T cells) in the various groups. **b**) the ratio of CD8^+^/CD4^+^ T cells in CD3^+^ T cells. **c**) Representative flow cytometry results of effector memory T cells, gated on CD4^+^. **d**) The frequencies of effector (CD44^hi^CD62L^low^) memory CD4^+^ T cells in the splenocytes. **e**) Representative flow cytometry results of effector memory T cells, gated on CD8^+^ T cells. **f**) The frequencies of effector (CD44^hi^CD62L^low^) memory CD8^+^ T cells in the splenocytes. Error bars represent ± SD (*n* = 6). The significance was analyzed using a one-way analysis of variance (ANOVA). *p < 0.05, **p < 0.01, ***p < 0.001, ****p < 0.0001

Encouraged by the results of the above in vitro and in vivo experiments, the ability of different nanovaccines to induce an antigen-specific immune response was further evaluated with the vaccination regimen shown in Fig. 5a. As mentioned earlier, DC activation is an important step to activate the natural immune response. After three immunizations against C57BL/6 mice, the activation level of DCs in the collected spleen was examined. As shown in Fig. 5b-g and Supporting Information Figure S8 (flow cytometry gating strategy was shown in Supporting Information Figure S7), the expressions of MHC II, CD80, CD86, and CD40 molecules were significantly promoted by the A-T base pair pairing group (MnO_2_/OVA/A_50_-T_20_) compared with OVA group, indicating that the MnO_2_/OVA/A_50_-T_20_ vaccine formulation significantly enhanced the maturation of DCs in vivo and induced more effective antigen delivery and immune response in immunized mice. Moreover, compared with the OVA group, the MHC I expression was significantly enhanced in the MnO_2_/OVA/A_50_-T_20_ group (Fig. 5d&f), which also verified that the MnO_2_/OVA/A_50_-T_20_ group had the highest cross-presentation efficiency, which could be attributed to the aggregation of nanoparticles after base pairing, increasing the chances for DCs to capture antigens and facilitate their delivery into the cytoplasm. The co-stimulatory molecule CD40 effectively promoted the formation of immune synapses between APCs and T cells, which in turn activated T cells, as shown in Fig. 5e. CD40 signaling is an important trigger for the monocyte maturation process, and binding of CD40 to the DCs surface promotes the production of cytokines and chemokines, induces the expression of co-stimulatory molecules, and facilitates antigen cross-presentation [37]. The expression of CD40 was much higher in the MnO_2_/OVA/A_50_-T_20_ group than in the MnO_2_/OVA and MnO_2_/OVA/A_50_ groups, which again suggests that A-T base pair pairing induced nanoparticle aggregation and then induced stronger body immunity. As shown in Fig. 6a&b, DCs activated by the MnO_2_/OVA/A_50_-T_20_ group vaccine formulation significantly increased the proportion of CD8^+^ T cells, implying that the vaccine effectively stimulated the proliferation and differentiation of T cells, suggesting its potential application as a therapeutic tumor vaccine for tumor immunotherapy.

### In vivo evaluation of the immune memory effect

The main function of vaccines in the natural immune system is to induce antigen-specific immune memory so that the immune system can respond rapidly when it is invaded by the same antigen. In this process, memory T cells are the key to generate effective immune memory in the body. Usually, memory T cells are divided into effector memory T cells (T_EM_, CD44^high^CD62L^low^) and central memory T cells (T_CM_, CD44^high^CD62L^high^) according to the cell surface receptors. Among them, T_CM_ can proliferate and differentiate into T_EM_ in response to antigen stimulation, while CD4^+^ T_EM_ can activate the immune system by inducing cytokine secretion such as INF-γ, and CD8^+^ T_EM_ can directly recognize and kill tumor cells. As shown in Fig. 6c-d and e-f, the MnO_2_/OVA/A_50_-T_20_ group was able to significantly induce a higher proportion of CD4^+^ T_EM_ and CD8^+^ T_EM_ compared to the other vaccine formulation groups, which further demonstrated that the MnO_2_/OVA/A_50_-T_20_ vaccine formulation induced more significant immune memory effects, indicating its potential role in tumor prevention that specifically kills target cells without destroying normal cells.

### In vivo antitumor efficacy

**Fig. 7:**
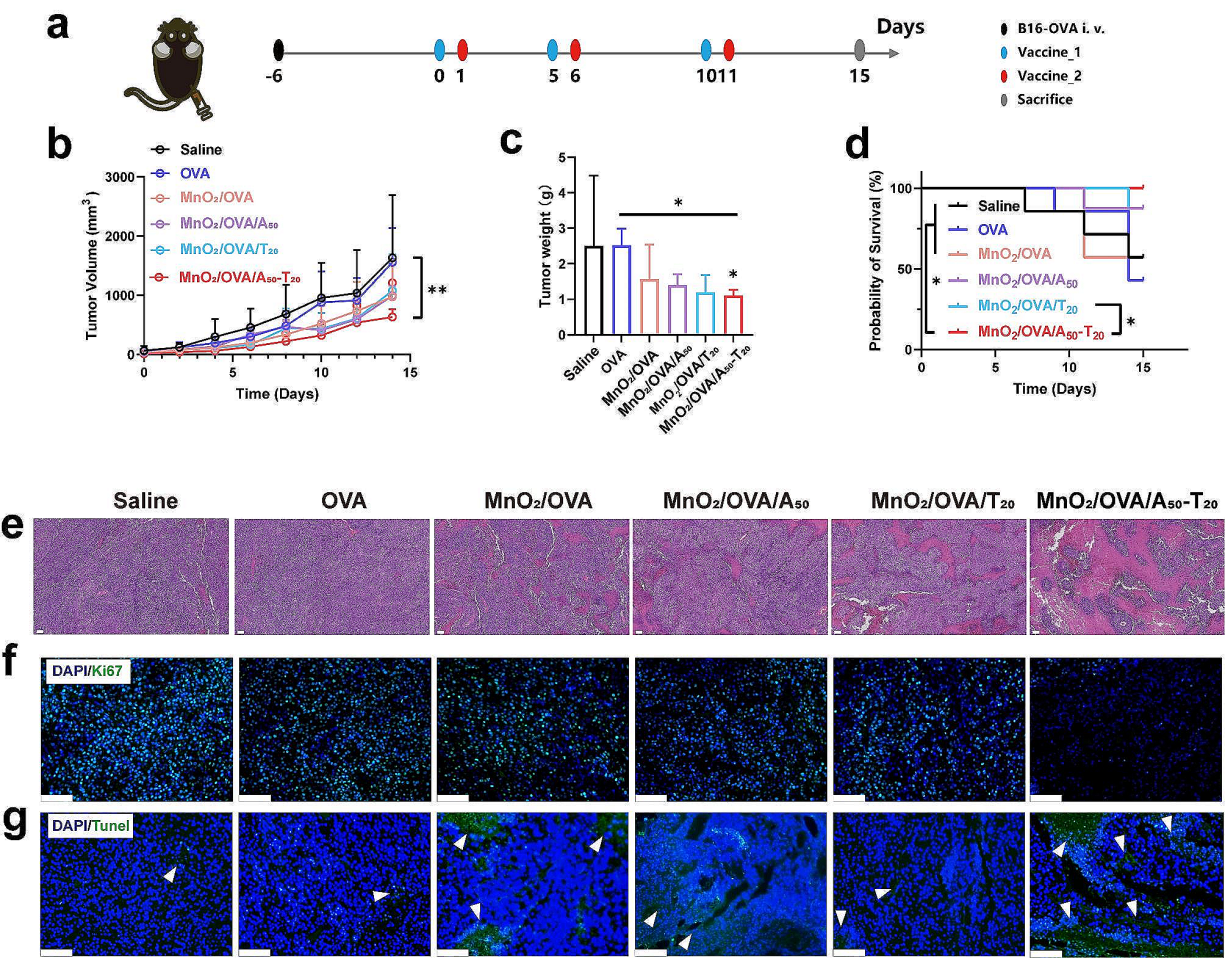
In vivo inhibition of melanoma tumors by treatment with nanovaccines. **a**) The schematic illustration of the tumor therapy experiment. **b**) Tumor growth curves of B16-OVA-tumor-bearing mice after various treatments. **c**) Tumor weights with various treatments collected on day 15. **d**) Mice survival time results. **e**) H&E images of tumor tissues collected on day 15 after various treatments. Immunofluorescence staining of **f**) Ki67 and **g**) Tunnel in the tumors. Error bars represent ± SD (*n* = 5). The significance was analyzed using a one-way analysis of variance (ANOVA). *p < 0.05, **p < 0.01, ***p < 0.001, ****p < 0.0001

To investigate the anti-tumor ability of those nanovaccines, we performed an in vivo anti-tumor assessment using the B16-OVA melanoma model. As shown in Fig. 7a, B16-OVA cells were injected subcutaneously in C57BL/6 mice, and after 7 days they were divided into six groups and inoculated subcutaneously with various agents on days 0, 1, 5, 6, 10, and 11. Tumor volumes were measured every other day during the treatment period, as shown in Fig. 7b and Supporting Information Figure S9. The average and individual tumor growth curves showed that the B16-OVA tumors grew rapidly in saline and OVA groups, and the nanovaccines significantly inhibited the tumor growth, with the most excellent tumor inhibition in the MnO_2_/OVA/A_50_-T_20_ group. Supporting Information Figure S10 showed that all groups of mice showed a slight increase in body weight with time. Tumor volume up to 1500 mm^3^ was used as the survival criterion, and only the survival rate of mice in the MnO_2_/OVA/A_50_-T_20_ group was maintained at 100% until the end of the antitumor experiments (Fig. 7c). Similarly, the tumor weight in the MnO_2_/OVA/A_50_-T_20_ group was slighter, which also confirmed that the MnO_2_/OVA/A_50_-T_20_ group had the best anti-tumor effect (Fig. 7d). Hematoxylin-eosin (H&E) staining of the tumor tissues showed that the tumors in the MnO_2_/OVA/A_50_-T_20_ group showed extensive necrosis, whereas the tumors in the other groups remained more viable (Fig. 7e). The immunofluorescence images of TUNEL (apoptosis) and Ki67 (cell proliferation), as illustrated in Fig. 7f and g, respectively, demonstrated that the MnO_2_/OVA/A_50_-T_20_ vaccine formulation induced more pronounced apoptosis and a larger area of damage than the other vaccine formulations under identical conditions [13]. Moreover, the extent of cell proliferation in the MnO_2_/OVA/A_50_-T_20_ group was significantly diminished, collectively indicating the favorable anti-tumor efficacy of the MnO_2_/OVA/A_50_-T_20_ vaccine formulation.

**Fig. 8:**
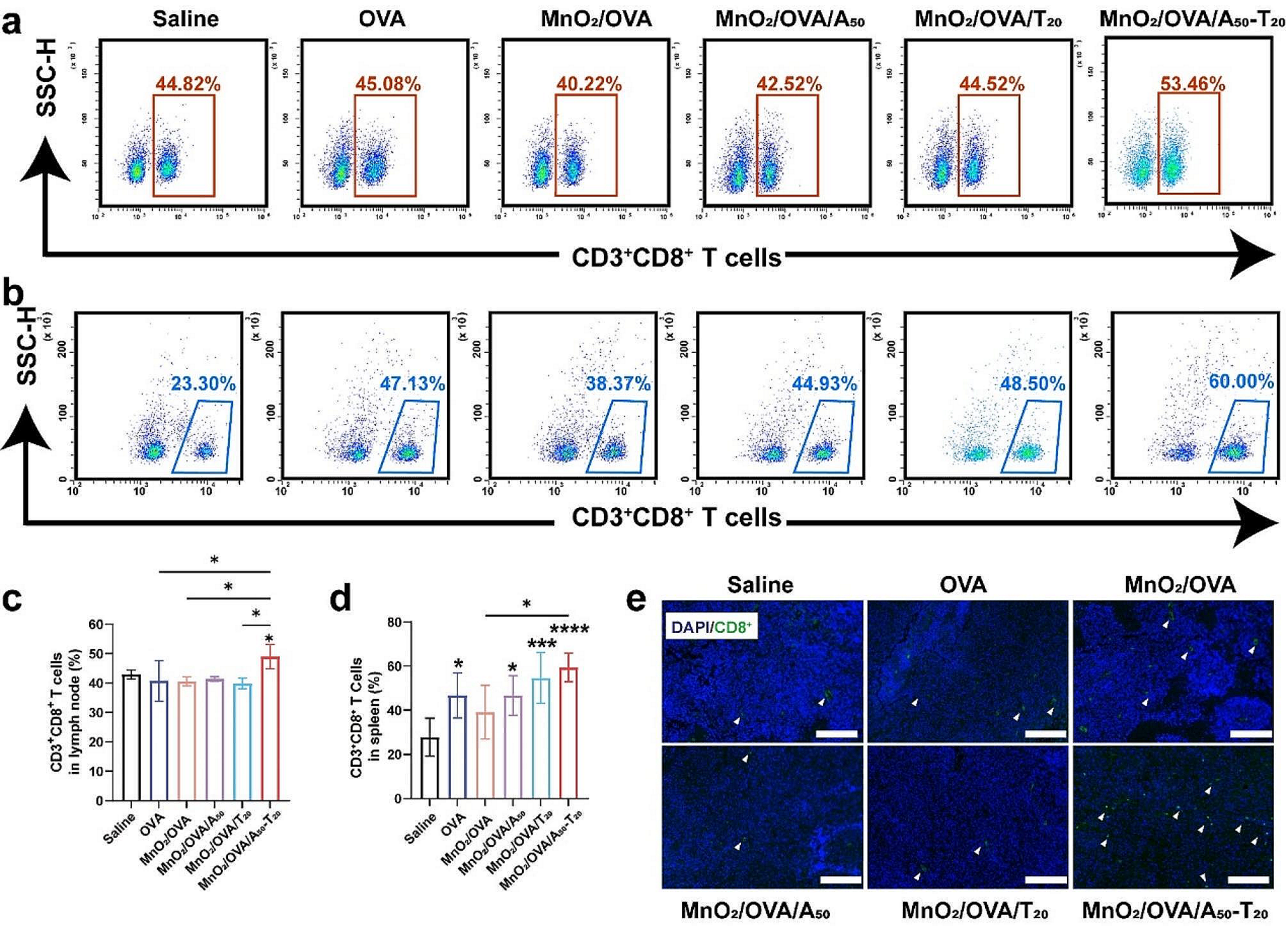
In vivo antitumor immunotherapeutic study. Representative flow cytometry results of **a**) CD3^+^CD8^+^ T cells in the lymph nodes and **b**) CD3^+^ CD8^+^ T cells in the spleens. **c**) The proportion of CD3^+^CD8^+^ T cells in the lymph nodes determined by flow cytometry. **d**) The proportion of CD3^+^CD8^+^ T cells in the spleen determined by flow cytometry. **e**) Immunofluorescence staining of CD8^+^ T cells in the tumors. Error bars represent ± SD (*n* = 5). The significance was analyzed using a one-way analysis of variance (ANOVA). *p < 0.05, **p < 0.01, ***p < 0.001, ****p < 0.0001

To investigate the in vivo immune response of the B16-OVA melanoma model, the inguinal LNs were collected from mice in different vaccine formulation groups, as shown in Supporting Information Figure S11. The volume of LNs in the DNA-containing vaccine formulation groups was significantly increased compared with MnO_2_/OVA groups, among which, the volume of LNs in the MnO_2_/OVA/A_50_-T_20_ group was the largest. These results suggested that a stronger immune response induced lymphocyte proliferation in the mice treated with the MnO_2_/OVA/A_50_-T_20_ group. CTLs, as the anti-tumor mainstay of the initial T cells differentiated after cellular immunity was activated. Here, single-cell suspensions of LNs and spleens were obtained by grinding, and the proportions of CTLs in the LNs and spleens were analyzed. As shown in Fig. 8a and c, the cytotoxic CD8^+^ T cells were significantly increased in the LNs of the mice treated with the MnO_2_/OVA/A_50_-T_20_ group. Their proportions in splenocytes were further explored as shown in Fig. 8b and d, compared with the saline group, the percentage of CD8^+^ T cells in the splenocytes of mice treated with the DNA-containing group was significantly higher, and the highest CD8^+^ T cells were found in the MnO_2_/OVA/A_50_-T_20_ group, which indicated that MnO_2_ as an adjuvant and nanoparticle aggregation due to A-T base pair pairing indeed assist in the activation of organismal immunity, especially the cellular immune response. T-lymphocyte infiltration in tumor tissues was equally important for tumor cell killing. The immunofluorescence staining of tumor tissue sections was then performed as shown in Fig. 8e. The distribution of CD8^+^ T cells was significantly increased in the tumors treated with the MnO_2_/OVA/A_50_-T_20_ group, which indicated that this vaccine formulation promoted CD8^+^ T cell infiltration of tumors and significantly exerted an anti-tumor immune response.

**Fig. 9:**
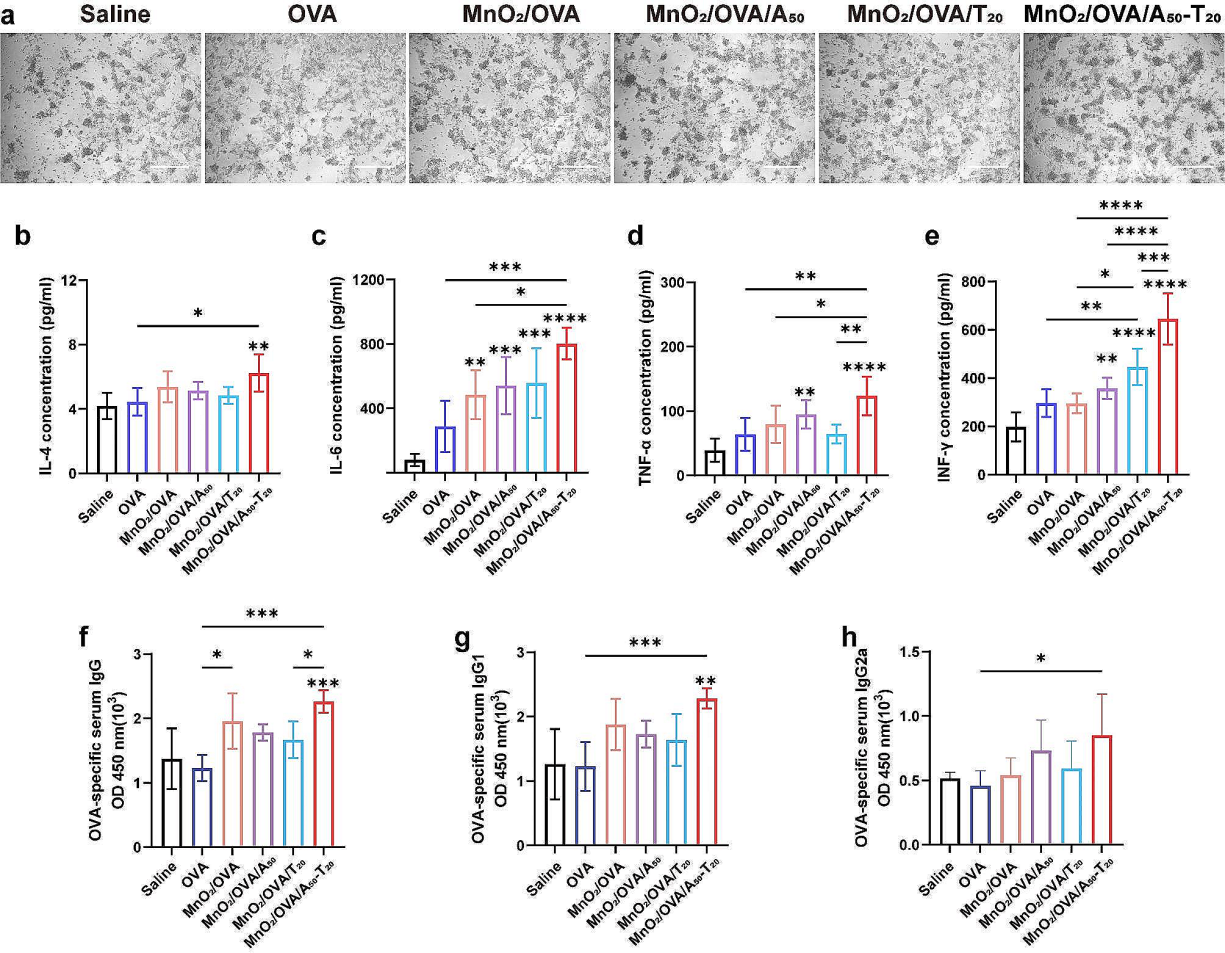
**a**) Photographs of tumor cell-specific killing studies. Scale bars: 250 μm. The serum nanovaccines-specific. **b**) Secreted levels of IL-4, **c**) IL-6, **d**) TNF-α, and **e**) INF-γ form splenocytes after co-culture with OVA. **f**) IgG, **g**) IgG1, and **h**) IgG2a titers on Day 15. Error bars represent ± SD (*n* = 5). The significance was analyzed using a one-way analysis of variance (ANOVA). *p < 0.05, **p < 0.01, ***p < 0.001, ****p < 0.0001

Subsequently, the collected splenocytes of the immunised mouse were further co-cultured with B16-OVA cells in vitro for 24 h, as shown in Fig. 9a. A large number of splenocytes in the MnO_2_-containing group were attached to the B16-OVA, and the splenocyte clusters of the MnO_2_/OVA/A_50_-T_20_ group were most obviously attached to tumor cells, which may be that the CD8^+^ T cells in the splenocytes exerted a specific toxicity to B16-OVA [38]. This also proves that the number of CD8^+^ T cells in splenocytes was the highest in the MnO_2_/OVA/A_50_-T_20_ group. In addition, the supernatants of splenocytes after co-stimulation with OVA antigen were collected for cytokine secretion level assays. As shown in Fig. 9b-e, compared with the saline group, the secretion of IL-6 cytokine was significantly increased in the MnO_2_-containing groups, and that was the highest in the MnO_2_/OVA/A_50_-T_20_ group. Meanwhile, compared with the saline and OVA group, the secretion of IL-4, IL-6, TNF-α, and INF-γ cytokines was significantly increased in the MnO_2_/OVA/A_50_-T_20_ group. These results indicated that MnO_2_ acted as an adjuvant and could be induced aggregation by A-T base pair pairing at LNs, which promoted the secretion of anti-tumor cytokines and induced stronger immunity. As one of the key indicators of humoral and cellular immune levels, OVA-specific antibody titers were also examined by ELISA, and the results were shown in Fig. 9f-h. compared to the saline and OVA groups, the levels of activated IgG, IgG1, and IgG2a antibody titers in the MnO_2_/OVA/A_50_-T_20_ group were significantly increased, which suggested that antigen-loaded MnO_2_ could effectively promote antibody production, and the vaccine further enhanced the production of cellular immunity-associated antibody after aggregation in LNs by base-pairing.

Considering the importance of biosafety for vaccines, major organs were collected from the mice vaccinated three times with different vaccine formulations. Major organs such as the heart, liver, spleen, lung, and kidney were observed under the microscope after H&E staining. As shown in Supporting Information Figure S12, no obvious abnormalities and lesions were observed in these organs of all groups, which indicated that the vaccine formulations prepared in the present study did not damage the organs and were sufficiently biologically safe.

## Conclusion

In conclusion, this study developed a size-adjustable vaccine delivery system based on the principle of base complementary pairing. This system was designed for targeted delivery to the LNs and to enhance the efficient uptake of antigens by APCs in LNs, thereby improving antigen delivery efficiency. Optimizing the nanoscale particle size of MnO_2_/OVA/A_50_ and MnO_2_/OVA/T_20_ (smaller than 100 nm), which could efficiently deliver them to the LNs. This optimization also induced the formation of nanoparticle aggregates within the LNs through DNA single-strand complementation, thereby increasing the retention time of vaccines in the LNs and facilitating the internalization of vaccines by APCs. These processes lead to effective APC activation, antigen cross-presentation, and a robust immune response in vivo, resulting in excellent anti-tumor effects. This study demonstrates that our vaccine inherits the advantages of traditional LNs-targeted vaccines while addressing potential drawbacks, such as insufficient internalization of the vaccine by APCs within the LNs. This work provides a validated strategy for targeting LNs and introduces innovative ideas for the development of more effective LNs-targeted vaccines.

### Electronic supplementary material

Below is the link to the electronic supplementary material.

Supplementary Material 1

## Data Availability

No datasets were generated or analysed during the current study.
