# Supplementary material for: Construction of lymph nodes-targeting tumor vaccines by using the principle of DNA base complementary pairing to enhance anti-tumor cellular immune response
Source: J Nanobiotechnology. 2024 May 8;22:230. doi: 10.1186/s12951-024-02498-1 (PMC11077755; doi:10.1186/s12951-024-02498-1)
Supplement: Supplementary file 1 — Supplementary Material 1 [file 12951_2024_2498_MOESM1_ESM.docx]

**Construction of lymph nodes-targeting tumor vaccines by using the principle of DNA base complementary pairing to enhance anti-tumor cellular immune response**

Yongchao Zha ^a, 1^, Li Fu ^a, 1^, Zonghua Liu ^a, *^, Jiansheng Lin ^b, *^, Linghong Huang ^a, *^

^a^ Department of Biomedical Engineering, Jinan University, Guangzhou, 510632, China

^b^ Department of Anatomy, Hunan University of Chinese Medicine, Changsha, China

Corresponding authors:

Linghong Huang, Email: tohlhong@163.com

Jiansheng Lin, Email: linjiansheng1020@163.com

Zonghua Liu, Email: tliuzonghua@jnu.edu.cn

^1^ These authors contributed equally to this work and should be considered as co-first authors.

**Supporting Figures S1-S12**

**
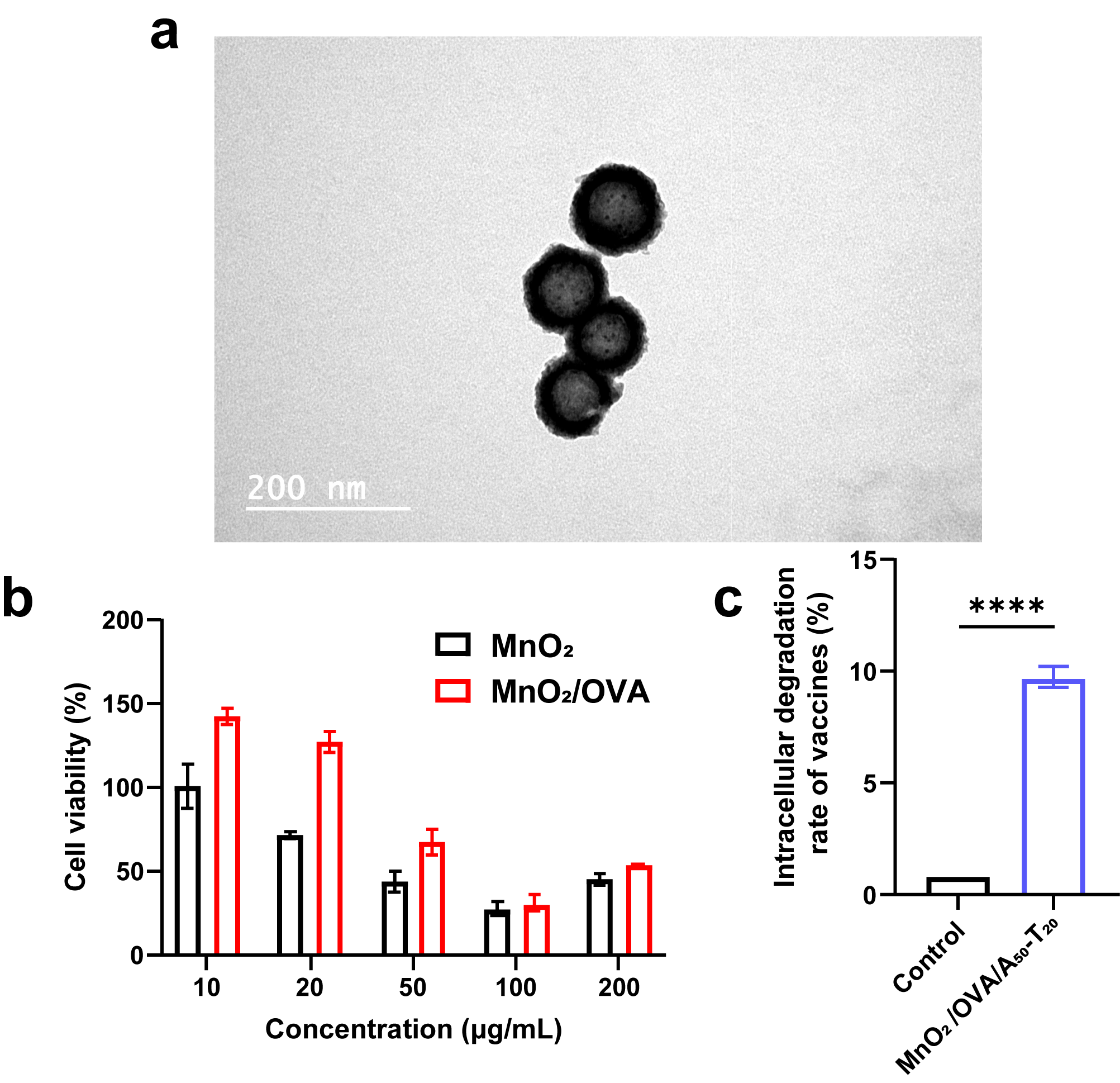
**

Figure S1. a) TEM of MnO_2_/OVA/DNA. b) Cytotoxicity assessment of MnO_2_ and MnO_2_/OVA incubated with DC2.4 cells for 24 h and measured by the CCK-8 assay kit. c) Intracellular degradation of nanovaccines.

**
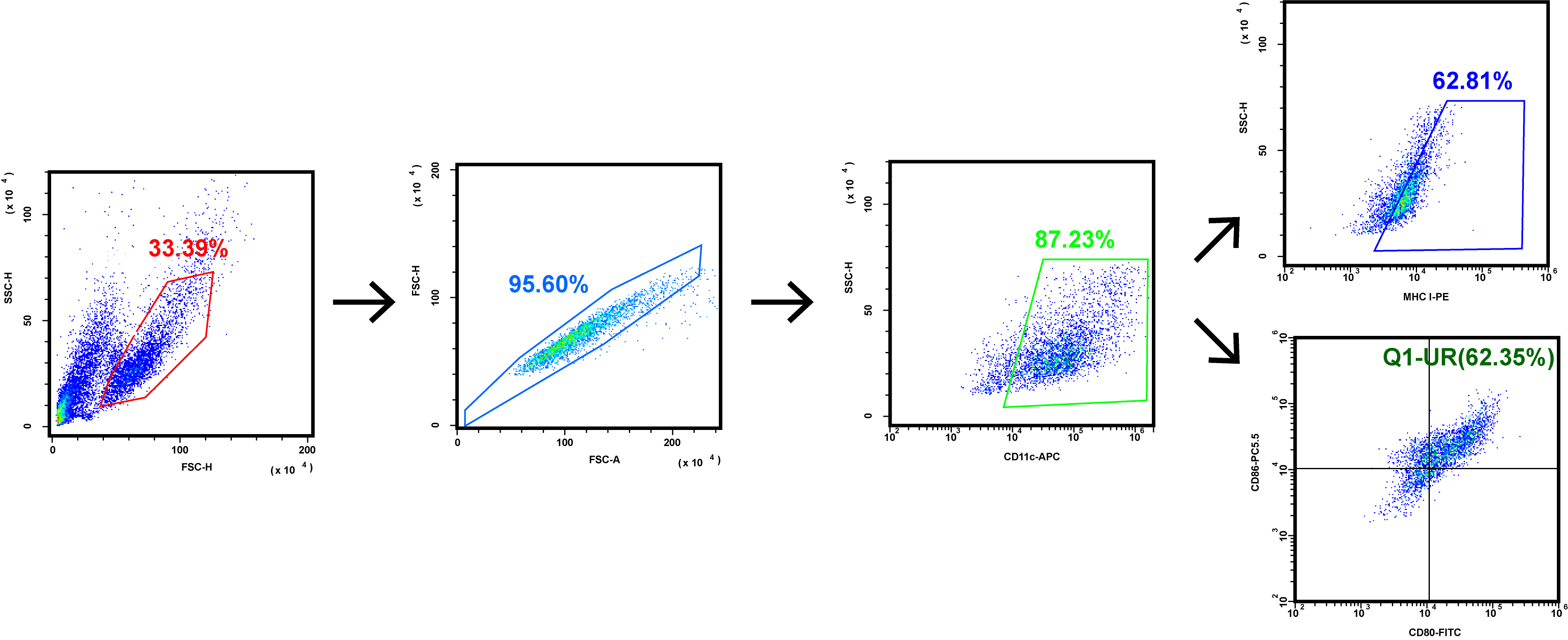
**

Figure S2. Flow cytometry gating strategy of CD80/86 and MHC-I in BMDC.

**
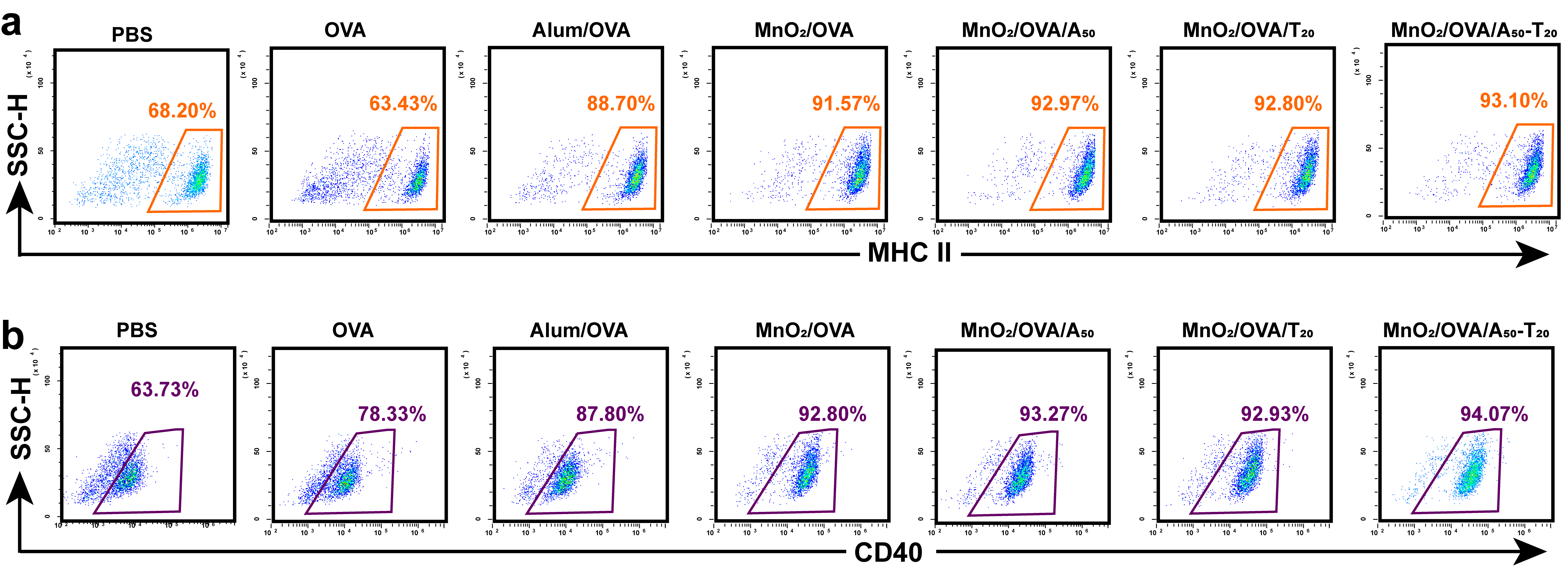
**

Figure S3. Representative flow cytometry dot plots of MHC II and CD40 (gated on CD11c^+^).

Figure S4. Secreted levels of INF-β from BMDC with or without MnO_2_.

**
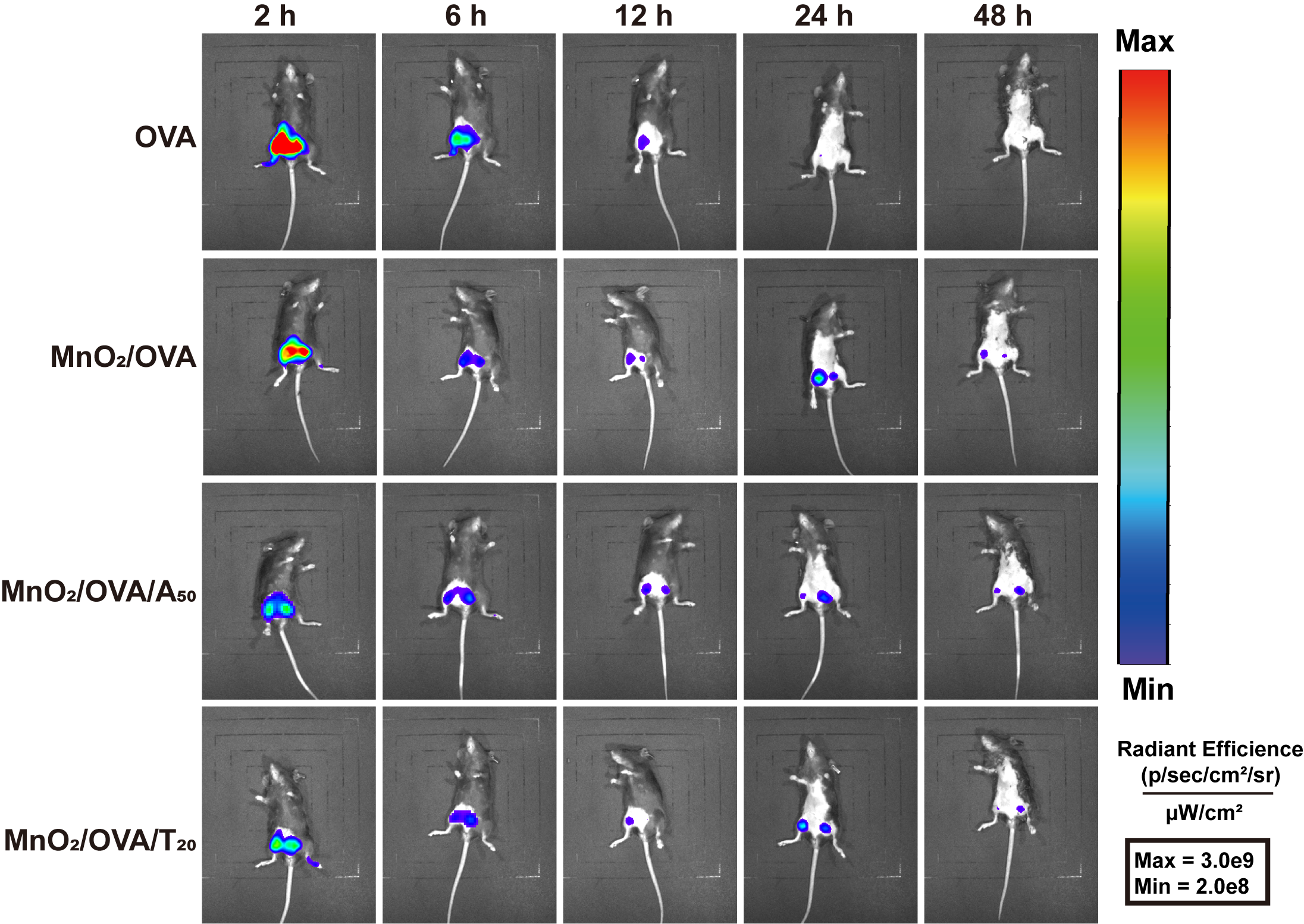
**

Figure S5. In vivo fluorescence imaging of mice (n = 3) at different times (2, 6, 12, 24, and 48 h) after subcutaneously injected with free OVA, MnO_2_/OVA, MnO_2_/OVA /A_50_, MnO_2_/OVA/T_20_. OVA was labeled with Cy5.

**
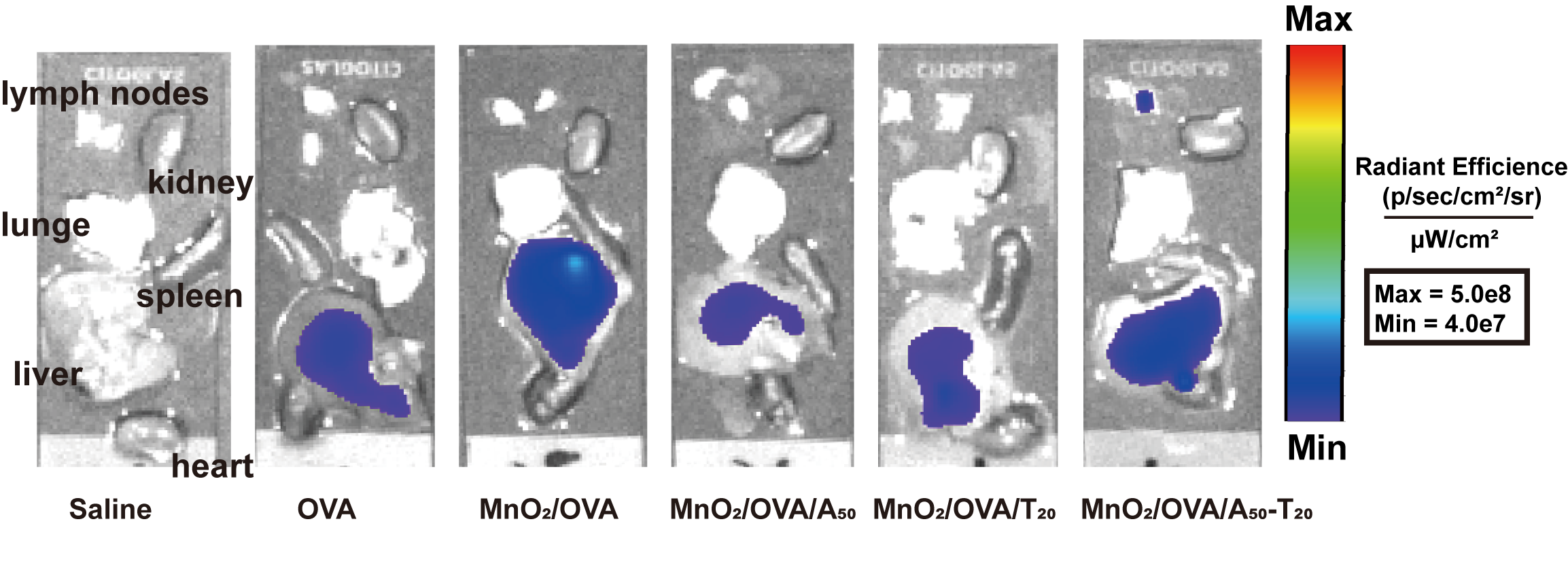
**

Figure S6. LN, kidney, lung, spleen, liver, and heart were excised from mice (n = 3) treated with various groups.


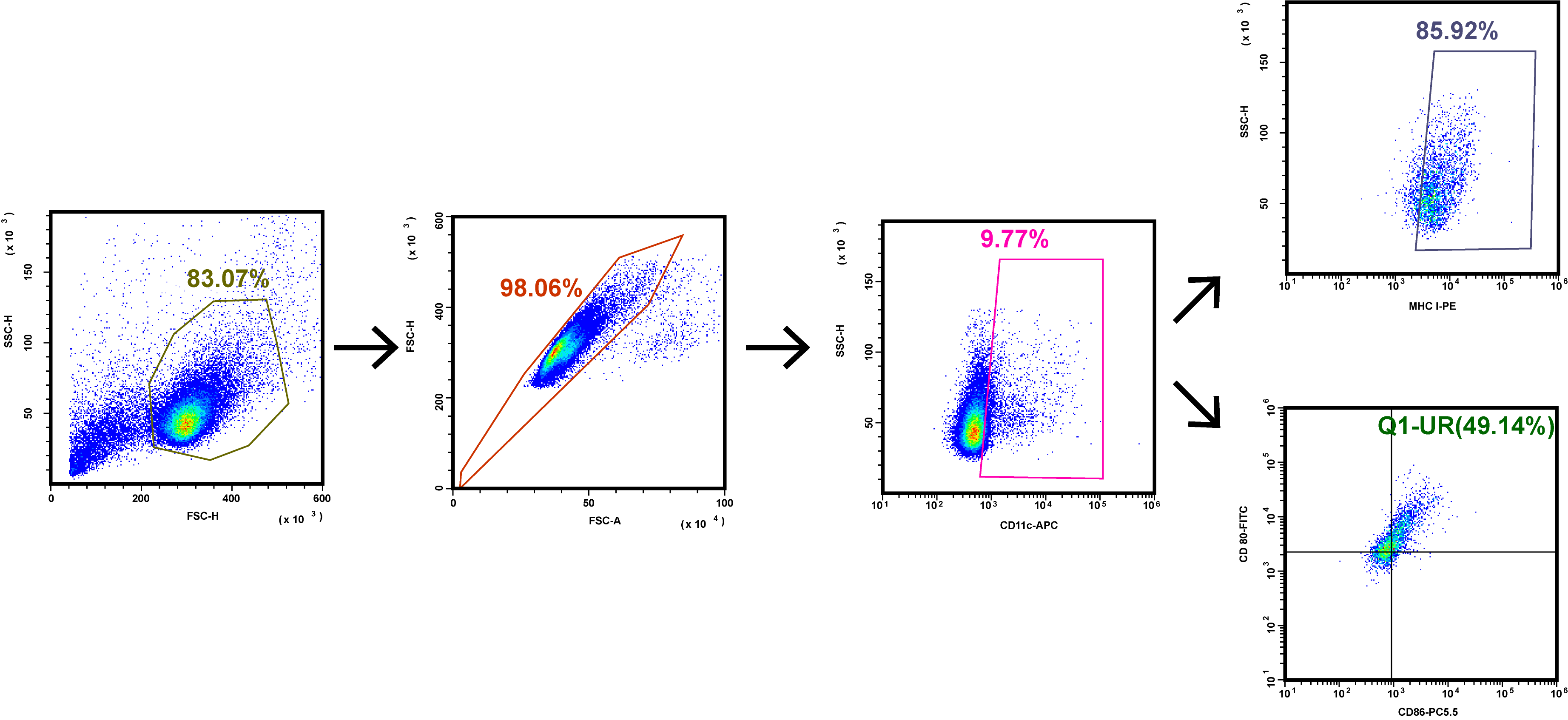


Figure S7. Flow cytometry gating strategy of CD80/86 and MHC-I in splenocytes.

**
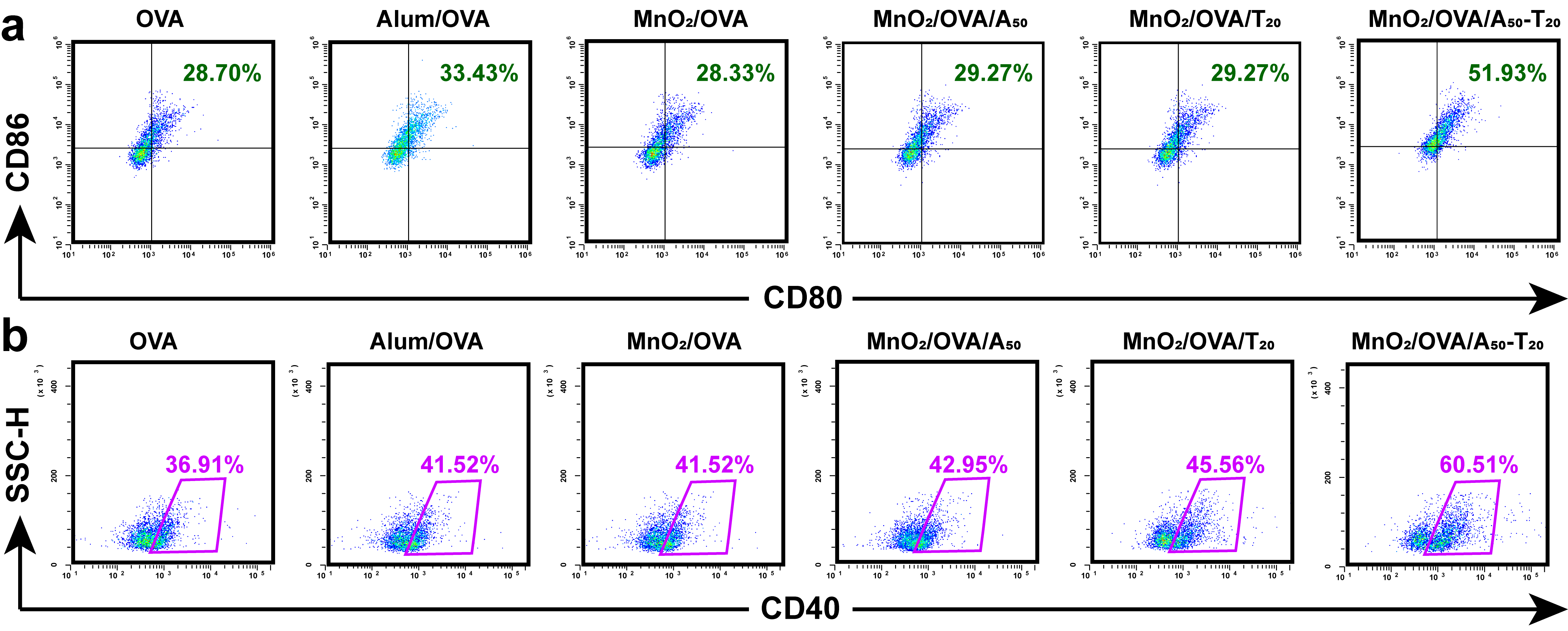
**

Figure S8. Representative flow cytometry dot plots of a) CD80^+^CD86^+^ and b) CD40 (gated on CD11c^+^).

**
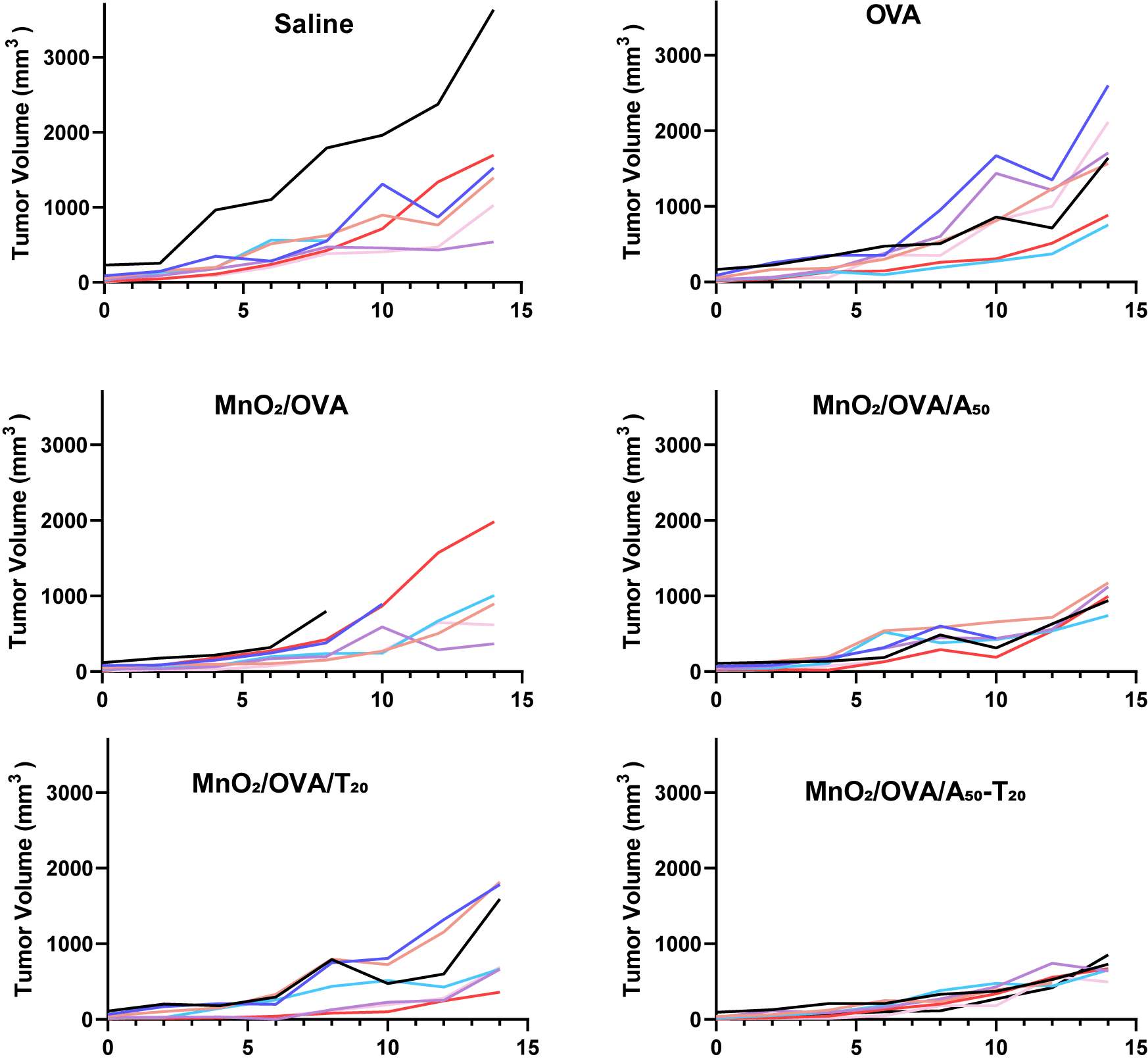
**

Figure S9. Average tumor growth curves for B16-OVA tumors in mice after various treatments.

**
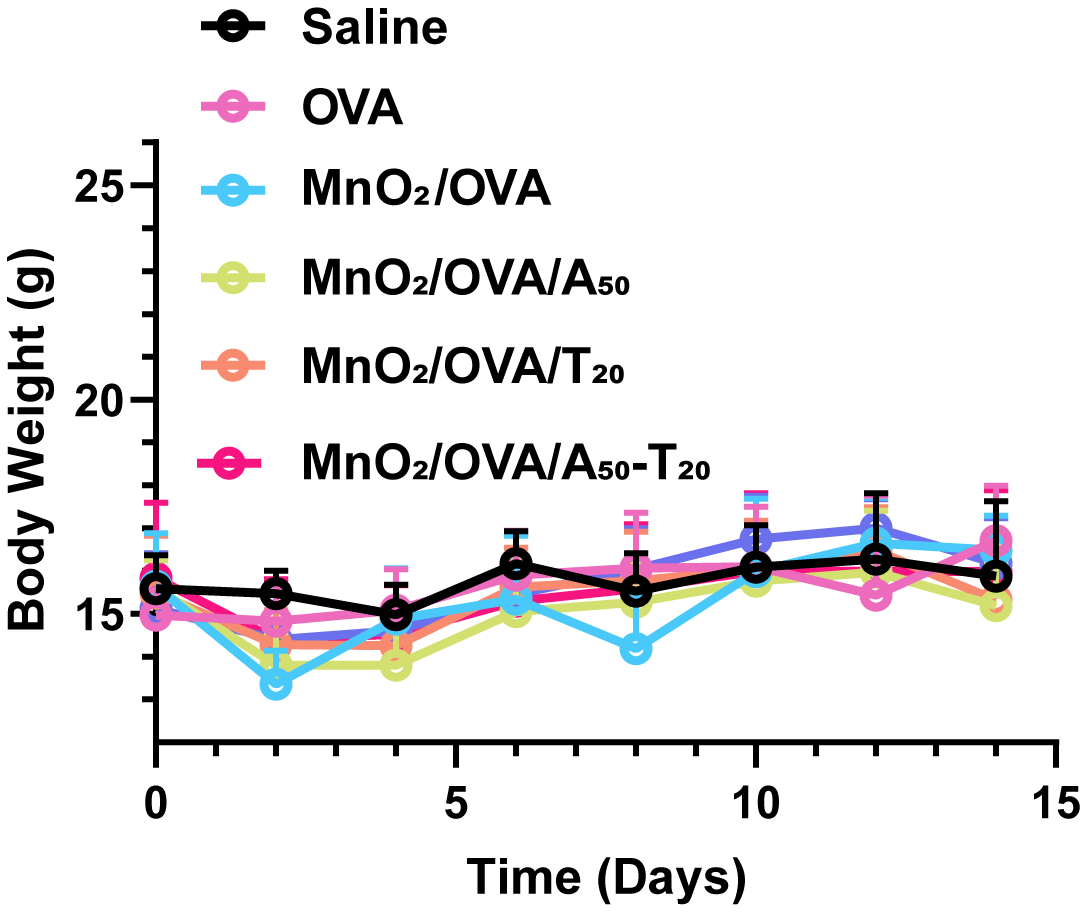
**

Figure S10. Body weight changes of mice in various groups during the therapeutic process.


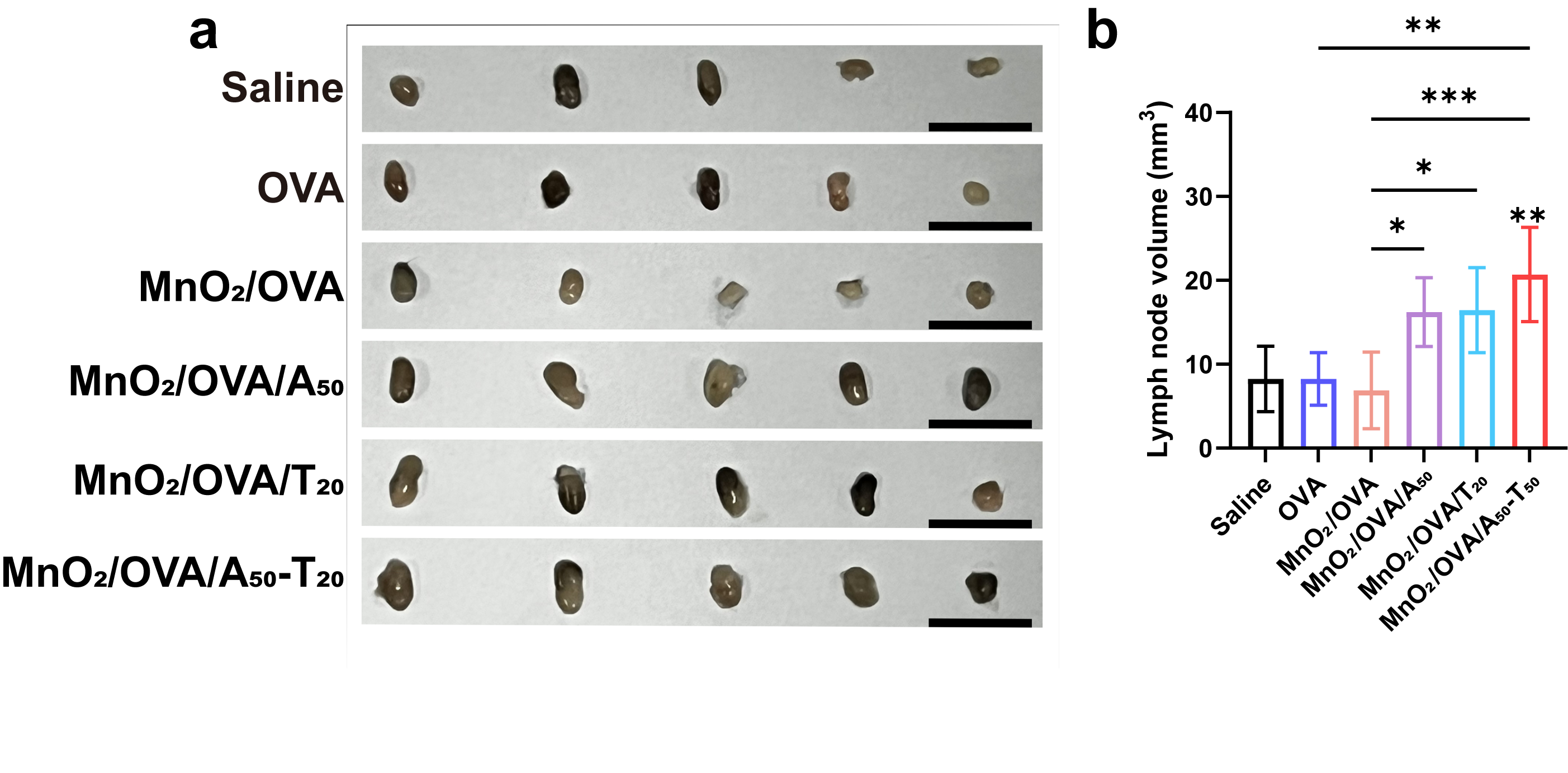


Figure S11. a) Photograph, scar bar: 1cm and b) lymph node volumes collected from mice.


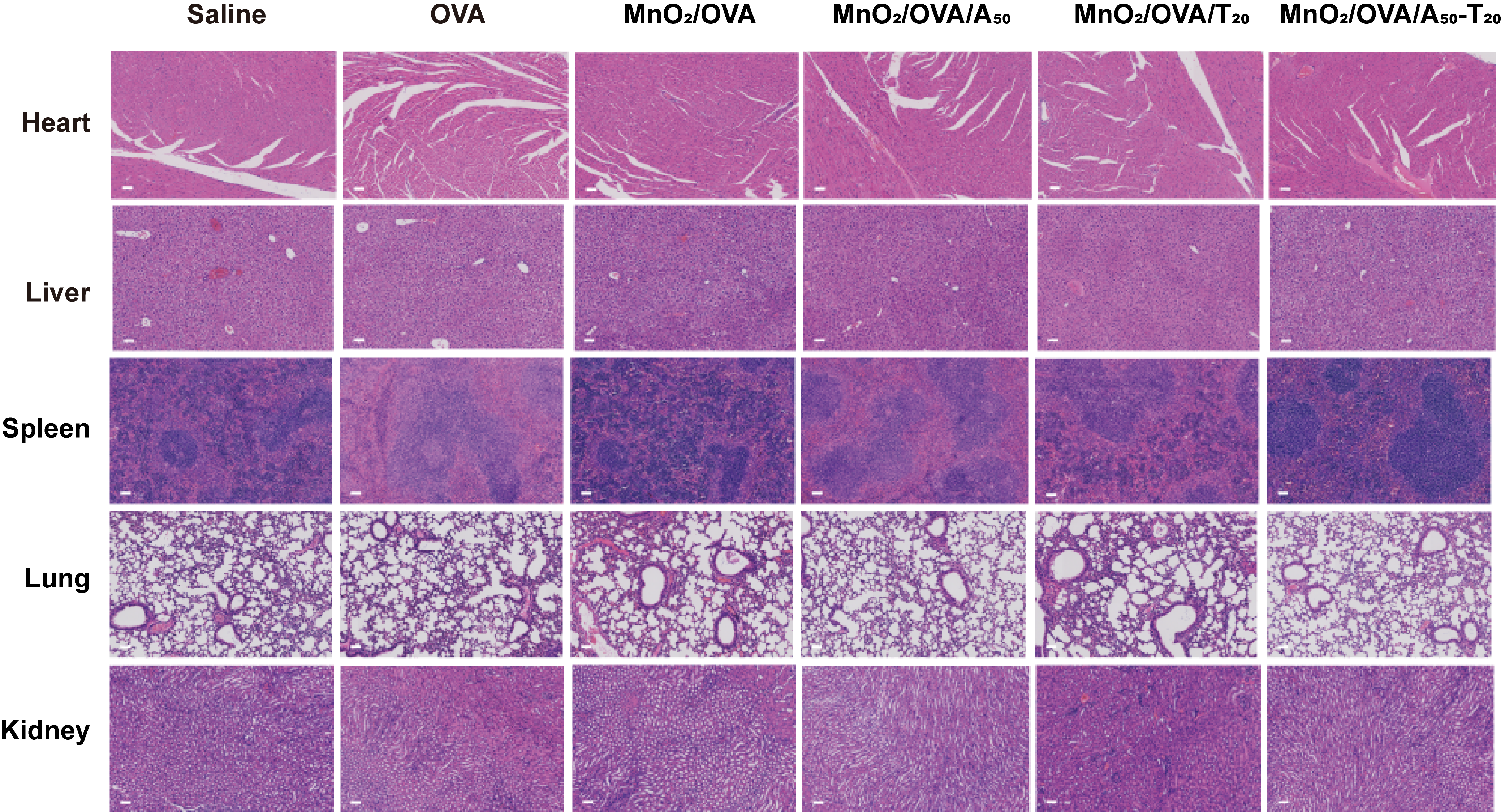


Figure S12. The H&E staining of major organs of each group of mice, scar bar: 100 μm.
